# Characteristic and Functional Analysis of Toll-like Receptors (TLRs) in the lophotrocozoan, *Crassostrea* *gigas*, Reveals Ancient Origin of TLR-Mediated Innate Immunity

**DOI:** 10.1371/journal.pone.0076464

**Published:** 2013-10-01

**Authors:** Yang Zhang, Xiaocui He, Feng Yu, Zhiming Xiang, Jun Li, Karen L. Thorpe, Ziniu Yu

**Affiliations:** 1 Key Laboratory of Marine Bio-resources Sustainable Utilization, Laboratory of Applied Marine Biology, South China Sea Institute of Oceanology, Chinese Academy of Sciences, Guangzhou, China; 2 Friedrich-Loeffler-Institut, Federal Research Institute for Animal Health, Institute of Immunology, Greifswald-Insel Riems, Germany; 3 Institute of Marine Sciences, University of Portsmouth, Portsmouth, United Kingdom; Virginia Tech University, United States of America

## Abstract

The evolution of TLR-mediated innate immunity is a fundamental question in immunology. Here, we report the characterization and functional analysis of four TLR members in the lophotrochozoans *Crassostrea* *gigas* (CgTLRs). All CgTLRs bear a conserved domain organization and have a close relationship with TLRs in ancient non-vertebrate chordates. In HEK293 cells, every CgTLR could constitutively activate NF-κB responsive reporter, but none of the PAMPs tested could stimulate CgTLR-activated NF-κB induction. Subcellular localization showed that CgTLR members have similar and dual distribution on late endosomes and plasma membranes. Moreover, CgTLRs and CgMyD88 mRNA show a consistent response to multiple PAMP challenges in oyster hemocytes. As CgTLR-mediated NF-κB activation is dependent on CgMyD88, we designed a blocking peptide for CgTLR signaling that would inhibit CgTLR-CgMyD88 dependent NF-κB activation. This was used to demonstrate that a *Vibrio parahaemolyticus* infection-induced enhancement of degranulation and increase of cytokines TNF mRNA in hemocytes, could be inhibited by blocking CgTLR signaling. In summary, our study characterized the primitive TLRs in the lophotrocozoan *C* *. gigas* and demonstrated a fundamental role of TLR signaling in infection-induced hemocyte activation. This provides further evidence for an ancient origin of TLR-mediated innate immunity.

## Introduction

Understanding the evolution of Toll-like receptor (TLR)-mediated innate immunity or ancient function of TLRs is an intriguing topic in immunology, due to their essential role in host defense of diverse organisms [1,2]. Mammalian TLRs play an important role in versatile recognition of pathogen-associated molecular patterns (PAMPs). On binding a PAMP, a TLR can initiate signaling transduction by recruiting its canonical adaptor Myd88 or others, leading to the activation of a NF-κB-mediated immune response. Similarly, in Drosophila Toll receptors contribute to the activation of NF-κB through the conserved adaptor Myd88; this is associated with an increased resistance to infection which has led to the suggestion that TLR-mediated innate responses share a common ancient ancestry. However, research on TLRs has revealed differences between insects and mammals in terms of domain organization, activation mode and function.

According to the number of CF motifs (cysteine cluster on the C-terminal end of LRRs), TLR families can be classified into two types: single cysteine cluster TLR (sccTLR) and multiple cysteine cluster TLR (mccTLR). All vertebrate TLRs belong to sccTLR, whereas most insect Tolls are mccTLR type with the exception of DmToll-9 which has only one CF motif [3,4]. Accumulated functional evidence also show that mammalian TLRs can be directly activated by the recognition of various PAMPs, such as TLR1, 2, 6 and 10 by lipopeptide, TLR3 by dsRNA, TLR4 by LPS, TLR5 by flagellin, and TLR7, 8 and 9 by nucleic acid and heme motifs [3,5]. In contrast, Drosophila Toll receptor does not directly recognize any PAMPs, but is instead activated by the endogenous ligand Spätzle [6]. Moreover, contrary to mammalian TLRs whose function is specific to innate immunity, Drosophila and *C. elegans* Tolls are also recognized as key regulators of embryonic development; this role is independent of NF-κB activation [7,8]. Thus, it is not clear whether the TLR-mediated innate immune response results from a common ancient ancestor or from independent co-option in different lineages.

Recent genomic data have shown that TLR genes are present in the genome of humans through to cnidarians, and that in most cases, they coexist with NF-κB signaling pathways (except nematodes), implying an ancient link between TLRs and innate immunity. However, the functional investigation of TLRs in invertebrates has been limited to two model organisms, *Drosophila* and *C. elegans*, both of which belong to ecdysozoans [9,10]. Considering the high diversification in function of TLRs and the lack of information regarding their role in species at key evolutionary positions, the origin of TLR-mediated innate immunity and ancient characteristics of TLRs remain uncertain. Additional information on the role of TLRs in other non-ecdysozoan invertebrates, such as photrochozoans, would therefore further our understanding of the origin and ancient characteristics of TLRs [11].

As the mollusk oyster *Crassostrea* *gigas* , is continuously exposed to marine water that is rich in microorganisms, it has evolved an effective innate immune system to cope with potentially harmful pathogens [12]. Evidence is also emerging that suggests oysters have a complete TLR and NF-κB signaling pathway, which plays a role in host defense against pathogen infection; this is considered as the functional analogue to human TLR and NF-κB [13-17]. Mollusks are a major lineage of lophotrochozoans and a sister branch of ecdysozoans, and may thus provide a key evolutionary step to understanding the ancient function and trace origin of the innate immune system. Functional information from lophotrochozoans may therefore further understanding of the evolution of TLR mediated innate immunity and the ancestral function of TLRs [11].

In this study, four TLR homologues are identified from *C* *. gigas* (CgTLRs), and their subcellular localization, ligand recognition and signaling adaptor investigated. Oyster specific TLR inhibitors, designed to provide a deeper insight into the *in vivo* function of TLR signaling in *C* *. gigas* , revealed that TLR signaling is linked to hemocyte activation and host immunity against bacteria. This provides the first functional evidence for TLR’s immune role in the lophotrocozoan and an ancient origin of the TLR-mediated innate immune response.

## Materials and Methods

### Ethics statement

All oysters used in the present study were marine-cultured animals and purchased from Nanshan fishery market of Qingdao (Shangdong Province, North China). No specific permits were required for oyster sample collection or described sampling. The location was not privately-owned or protected, and the field studies did not involve any endangered or protected species.

### Cloning the full-length cDNA of CgTLRs and CgMyD88

The homologues of TLR (AM858416, FP000363, AM854726 and FP002604) and MyD88 (FP002214) were identified using a BLAST (http://www.ncbi.nlm.nih.gov/blast) search against the Pacific oyster EST library. Based on these ESTs, gene specific primers (Table S1) were designed to amplify the full-length cDNA of CgTLRs and a MyD88 homologue (CgMyd88) by Rapid amplification of cDNA ends (RACE) technique using the GeneRacer^TM^ kit (Invitrogen, CA, USA). The amplified PCR products were cloned into pGEM-T Easy Vector (Promega, WI, USA) and sequenced with forward and reverse universal primers using ABI Prism 3730 DNA sequencer (PerkinElmer, Wellesley, MA, USA). Full-length cDNA sequences were obtained by combining the 3’- and 5’-end sequences. All of the sequences were deposited in GenBank under accession numbers from KC700617 to KC700620 for CgTLRs and KC700621 for CgMyd88.

### Bioinformatics analysis

Deduced amino acid sequences for CgTLRs were compared with previously published sequences for representative invertebrates and vertebrates. The protein domains were predicted with the Simple Modular Architecture Research Tool (SMART) version 4.0 (http://smart.embl-heidelberg.de/). The alignment of amino acid sequences was performed using ClutalX 1.81. The pairwise molecular distances were calculated using the MEGA 4.0 [18] based on the Poisson model. Multidimensional scaling (MDS) of the molecular distances was analyzed and presented using R-language.

### Plasmid construction

To assess the ability of CgTLRs to activate NF-κB transcriptional activity, expression vectors of CgTLR1-4 were constructed; the primers used are listed in Table S1. The target ORFs were amplified and inserted into pcDNA4.0 vector (Invitrogen, CA, USA) in corresponding restriction enzyme sites. Complete CgMyD88 and TIR domain (amino acids 1-237) deletion mutants of CgMyD88 (CgMyD88-TIR) recombinant vectors were also constructed. The NF-κB-dependent luciferase reporter vector including six tandems repeats of NF-κB binding sites and one minimal TA promoter was constructed in our laboratory [14]. Renilla luciferase pRL-TK vector (Promega) was used as an internal control. To test the intracellular localization of CgTLRs, four CgTLRs were inserted into pEGFP-N1 (invitrogen) to construct EGFP-tagged CgTLR vectors. Dsred-tagged Rab7 (constructed previously in our laboratory [19]) was used as a marker of late endosomes.

### Cell culture and transfection and NF-κB reporter assay

HEK293 cells were grown in Dulbecco’s modified Eagle’s medium (DMEM, Gibco) containing 10% heat-inactivated fetal bovine serum (FBS) and 10^5^ U/L penicillin and 100 mg/L streptomycin at 37 °C in a humidified incubator under 5% CO_2_.

Transfection was performed using Lipofectamine 2000 (Invitrogen) according to manufacturer’s instructions. Empty vector and CgTLR expression vectors with NF-κB-responsive reporter vector and pRL-TK internal control vector were, respectively, transiently transfected into HEK293 cells. Cells were collected and lysed at 24 h post transfection for detection of luciferase activities.

### Ligand stimulation and laser confocal imaging

All TLR ligands were purchased from InvivoGen and Sigma; details of the concentrations used are listed and summarized in Table 1. At 24 h post transfection, each PAMP was added to cell medium, and the cells incubated for an additional 12 h. Luciferase activities were expressed as the fold stimulation relative to empty vector-transfected cells incubated with the same stimulus.

**Table 1:** The applied TLR Ligands.

Ligand	Working Concentration	Recognized by Human TLR
Pam3CSK4, synthetic tripalmitoylated lipopeptide	1µg/ml	TLR1/2
HKLM, heat-killed *Listeria monocytogenes*	10^8^ cells/ml	TLR2
PGN, peptidoglycan from *Bacillus subtilis*	1µg/ml	TLR2
Poly I:C, synthetic analog of double-stranded RNA	1µg/ml	TLR3
LPS, Lipopolysaccharide from *E. coli K12*	1µg/ml	TLR4
HKVP, heat-killed *Vibrio Parahaemolyticus*	10^8^ cells/ml	
Flagellin from *S* *. typhimurium*	1µg/ml	TLR5
FSL-1, synthetic lipoprotein	1µg/ml	TLR6/2
Imiquimods, imidazoquinoline amine analogue to guanosine	1µg/ml	TLR7
ssRNA, single-stranded RNA oligonucleotide	1µg/ml	TLR8
ODN2006, synthetic oligonucleotides containing unmethylated CpG dinucleotides	5µM	TLR9
1,3-β-glucan from *Saccharomyces cerevisiae*	1µg/ml	TLR2
Larminarin from *Laminariadigitata*	1µg/ml	

HEK293T cells were seeded onto coverslips and transfected with EGFP-tagged CgTLR vectors. At 24 h post-transfection, cells on the coverslips were washed once with PBS, and then visualized using a laser scanning confocal microscope (Leica, Germany).

### Animal collection, primary culture of hemocytes and PAMPs challenge

The Pacific oyster, *C* *. gigas* , (two-years old with an average shell height of 90 mm) was obtained from Qingdao, Shangdong Province, China, and acclimatized at 22-25°C in tanks with circulating seawater for two weeks prior to use. Primary cultured hemocytes were prepared as previously described [20]. The cell viability of primary cultured hemocytes was assayed by the trypan blue (Sigma) exclusion technique. The mixed primary culture of hemocytes (3 ml in each plate) was challenged by adding 10 µg/ml polyI:C, 10 µg/ml LPS, 10 µg/ml PGN, 10 µg/ml 1,3-beta-glucan, 10^8^ cells/ml heat-killed *L. monocytogenes*(HKLM) and *V. Parahaemolyticus*(HKVP), respectively. Hemocytes from three replicates were harvested at different time points (3, 6, 12, 24 hours after challenge) for RNA isolation.

### Isolation of total RNA and quantitative real-time PCR

Total RNAs were extracted from tissues with TRIzol Reagent (Invitrogen, USA) according to the manufacturer’s directions. The integrity of RNA was checked with 1.5% agarose gel electrophoresis. To synthesize cDNA, 500 ng of total RNA was used to performed Reverse transcription (RT) by PrimeScript^TM^ RT Reagent Kit Ver.2.0 (Takara Bio, Japan) following the manufacturer’s instructions.

The *CgTLRs and CgMyD88* expression patterns were determined by quantitative real-time PCR (qPCR) and normalized with three reference genes, namely elongation factor 1-alpha (EF1α), ribosome protein 1 (rp1) and glyceraldehyde-3-phosphate dehydrogenase (GAPDH). The primers used for the qPCR analysis are listed in Table S1; negative controls were also run to confirm that there was no genomic contamination. The qPCR was performed using a LightCycler 480 (Roche) with 20 µL volume including 10 µL of 2× Master Mix (Roche), 0.4 µL of each of the forward and reverse primers (10 mM), 1 µL of 1:10 diluted cDNA, and 8.2 µL of PCR-grade water. A dissociation curve analysis was performed at the end of each qPCR reaction to confirm specificity of amplicons. Each sample was carried out in triplicate, and expression levels of *CgTLRs and CgMyD88* were calculated using methods of multiple control, which normalized with geometric averaging of multiple reference genes as previously described [21].

### TLR inhibitor design and synthesis

The blocking peptide (BP) was designed based on BB loops of CgMyd88 TIR domain coupled with an N-terminal carrier in tandem as described previously [22]. The BP (RQIKIWFQNRRMKWKKIPWRDDLPGGSRYE) and control peptide (CP, RQIKIWFQNRRMKWKKSLHGRGDPMEAFII) were synthesized by Biosynthesis, purified by HPLC and their purity confirmed by mass spectrometry. The final peptide purity was 97-99%. The stocks of peptide (10 mM) were dissolved in 25% DMSO and kept frozen at -80°C. The required working solution was diluted with distilled deionized water.

### In vivo injection of TLR inhibitor and fluorescence-activated cell sorting (FACS) analysis

One group of oysters (n = 20) was injected with 100µL of the TLR blocking peptide (100 µM) into adductor muscles, and another group (n = 20) was injected with an equal volume of control peptide as a negative control. 12 h after injection, 10 oysters from each group were infected with *V. parahaemolyticus* as previously described [23], while the remaining 10 were maintained as controls. Hemolymph was then collected from the pericardial cavity through the adductor muscle using a syringe at 24h post-infection. Hemocyte populations in each group were measured using flow cytometry (BD FACSCalibur) based on FSC and SSC parameters; a total of 10000 events were acquired for each sample. The hemocytes population was calculated using Cell Quest Software.

### Statistical analysis

All data are presented as means ± S.D. Statistically significant differences were analyzed by one-way ANOVA, followed by Dunnett test (for comparing treatment groups with the control group) or Tukey multiple comparison test (for comparing all pairs of groups) using SPSS 13.0. Where only two groups were compared in a data set, Student’s t-test was used instead. Significance was set at p< 0.05.

## Results

### Characteristics and phylogenic analysis of CgTLR gene family

**Figure 1:**
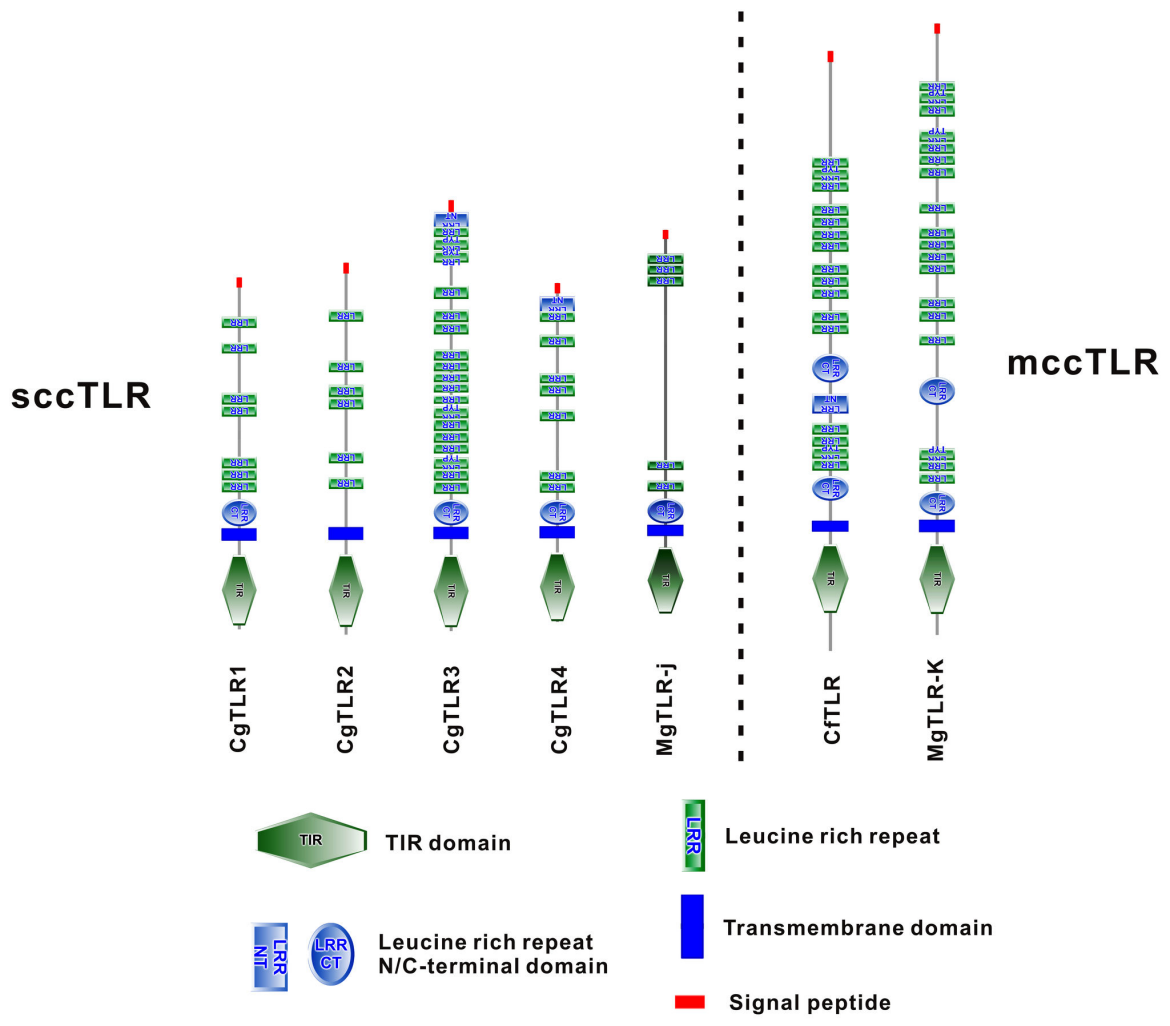
The structural organization of TLRs from *C* *. gigas* , *M* *. galloprovincialis* (MgTLR-j, KC357777 and MgTLR-k, KC357778) and *C* *. farreri* (CfTLR, ABC73693). Each of the four CgTLRs belongs to sccTLRs and contains a TIR domain, a TM domain, multiple extracellular LRRs and an N-terminal signal peptide.

Here, we obtained four full-length sequences of CgTLR cDNAs; the ORFs encoding putative proteins ranged from 697 to 876 amino acids in length (NCBI No. KC700617-KC700620, Figure S1). SMART analyses predicted that the four deduced CgTLR proteins bear an intracellular C-terminal TIR domain, a TM domain, multiple extracellular LRRs and an N-terminal signal peptide (Figure 1); designated here as CgTLR1 to CgTLR4, respectively. All members of the CgTLR family contain variation of LRRs from 6 to 14, arranged in tandem in extracellular region and flanked by a CF motif. Thus, all CgTLRs belong to sccTLRs rather than mccTLRs, such as CfTLR and MgTLR-k.

**Figure 2:**
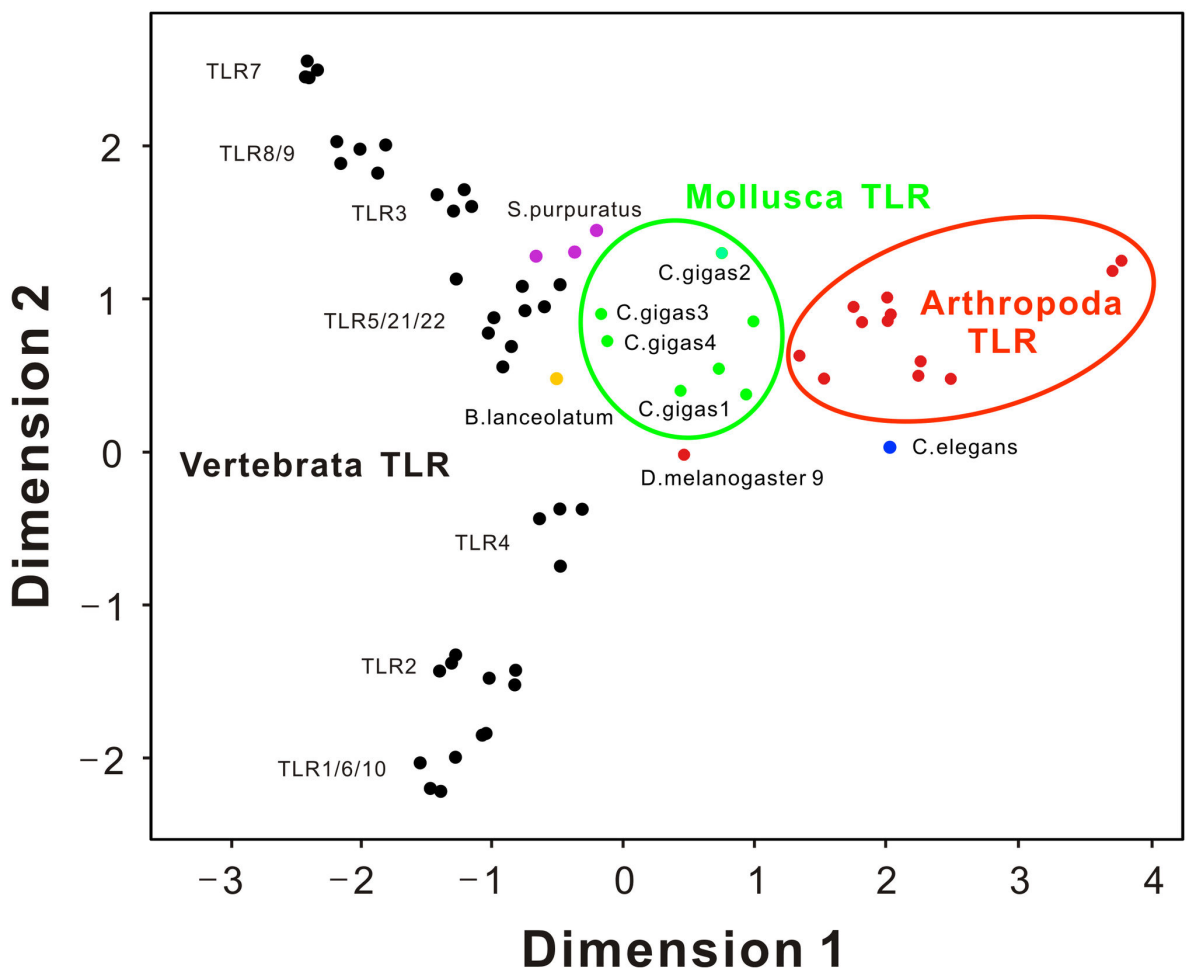
The MDS analysis of evolutionary relationships among chordata, mollusca and arthropoda TLRs. The sequences used for MDS analysis are listed in table S2. Different colors are used to distinguish between phylum species. The distance between any two points on the plot refers to their molecular distance or divergence. Mollusk and arthropod TLRs are much less divergent than that within vertebrata TLR family. The TLRs of ancient non-vertebrate chordates like *S. purpuratus* and *B* *. lanceolatum* is closer to CgTLRs than to arthropoda TLRs.

To illustrate the evolutionary relationships within the TLR family, we performed MDS to portray the relative molecular distances between these proteins. The results showed that the vertebrata TLR family has a much higher level divergence than that within mollusk and arthropod groups. Strikingly, the TLRs of ancient non-vertebrate chordates like *S. purpuratus* and *B* *. lanceolatum* is closer to CgTLRs than to arthropoda TLRs, strongly suggesting the pivotal positions of Cg-TLRs as prototypes of ancient TLRs (Figure 2).

### NF-κB reporter assay and response to PAMPs

**Figure 3:**
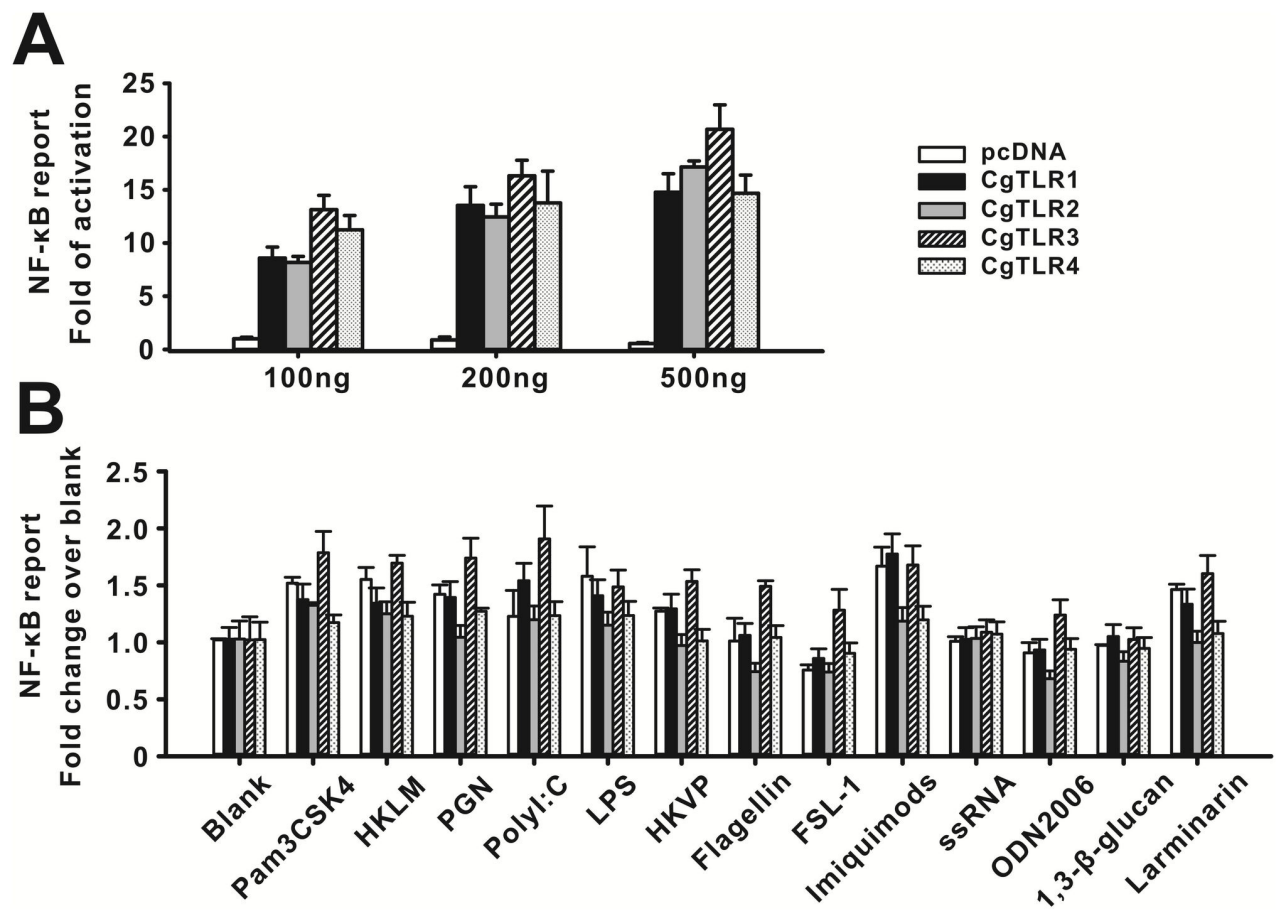
The NF-κB activity activated by CgTLRs. (A) The four CgTLRs can activate NF-κB-responsive reporter in a dose-dependent manner by transfection of plasmids increasing from 100 to 500 ng per well. HEK293 cells were transiently transfected with empty vector pcDNA or CgTLR expression vectors with 20 ng NF-κB-responsive reporter vector and 20 ng pRL-TK internal control vector per well in 24-well plate, respectively. Luciferase activities were tested at 24 h post transfection. (B) No PAMP can enhance higher induction of CgTLR-triggered activation of NF-κB. Transfected cells were then treated with 13 PAMPs, respectively. The groups without PAMP treatment were taken as blank. Luciferase activities were tested at 12 h post treatment. Each column and error bar represents the mean±S.D. (n = 5).

Mammalian TLRs can transactivate the transcriptional factor NF-κB in response to their specific ligands. Therefore, we used a NF-κB-responsive reporter assay to elucidate recognition ability of CgTLRs to PAMPs. Our results showed that each CgTLR can activate NF-κB responsive reporter in HEK293 cells (n = 5, p < 0.001; Figure 3A), and stronger activations were seen when transfections of CgTLR expression vectors increased from 100 ng to 500 ng per well. Transfected cells were then treated with 13 PAMPs at concentrations that could trigger significant activation of the respective human TLRs, however, there was no evidence that PAMP elicit significant responses in CgTLR expression vector transfected cells. TLR ligands poly I:C, P3CSK4 and FSL-1could induce a 1.5-fold increase in NF-κB transactivation in CgTLR3 transfected cells, however this increase was not significant when compared with blank group (Figure 3B).

### Cellular localization of CgTLRs

**Figure 4:**
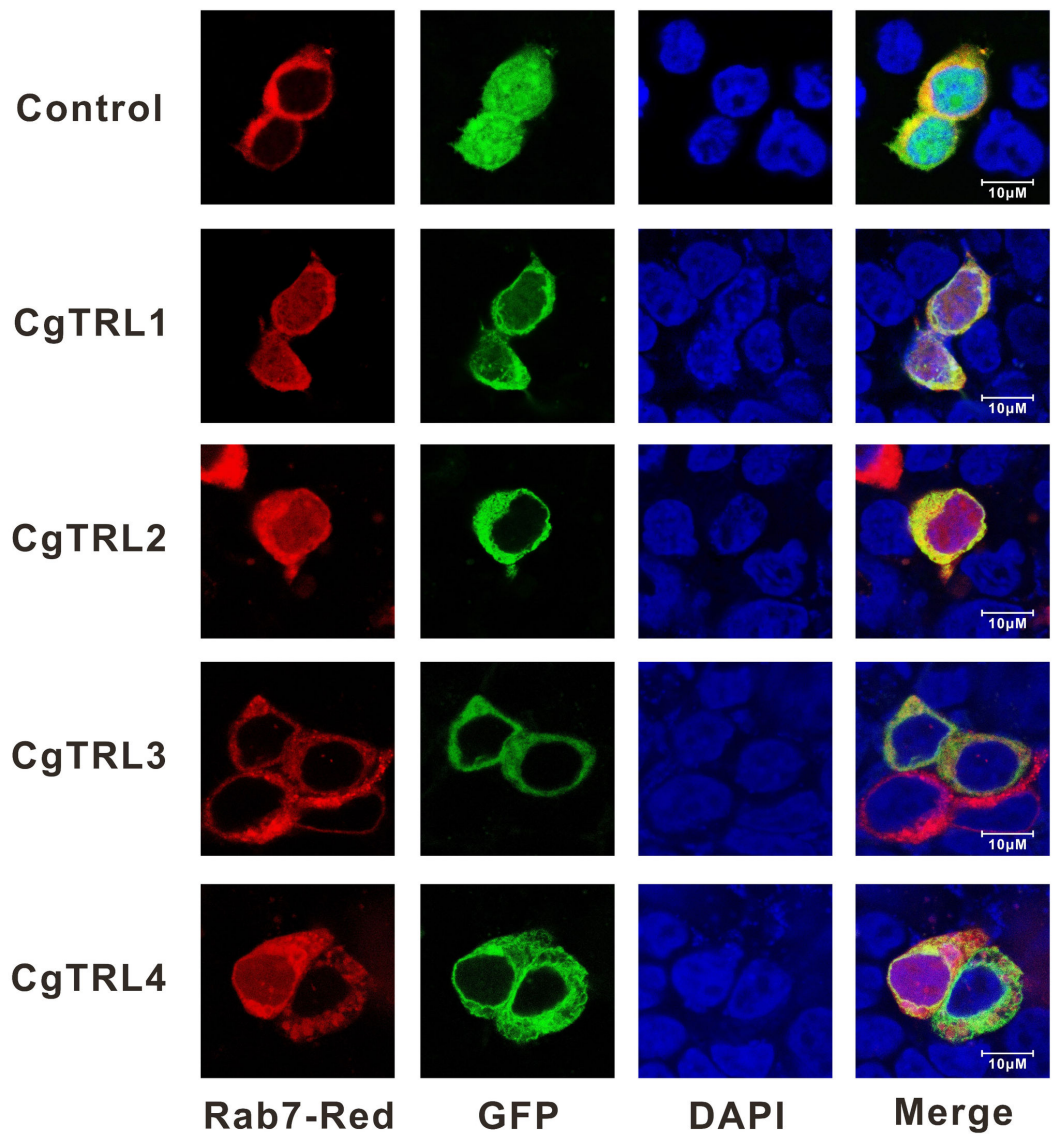
Subcellular localization of CgTLRs in HEK293 cells. Empty plasmid pEGFP-N1was used as control. Red, green and blue colors show localization of Rab7-Red, the GFP tag or GFP-tagged CgTLRs and nucleus, respectively. Rab7-Red was a marker for late endosome. The four CgTLRs have similar intracellular localizations, majoring on late endosomes and minoring on plasma membranes.

Confocal microscopy observations of the GFP-tagged CgTLR (expressed in HEK293 cells) revealed that the EGFPs fusion proteins of CgTLR are primarily located in the cytoplasm and membrane (Figure 4). In contrast, the cell harboring the negative control plasmids pEGFP-N1 showed a uniform presence of green ﬂuorescence throughout the cell. Further, Dsred-tagged Rab7 staining showed that the two fluorescences largely merged together, suggesting localization of endosomes.

### CgTLRs association with MyD88 to activate NF-κB

**Figure 5:**
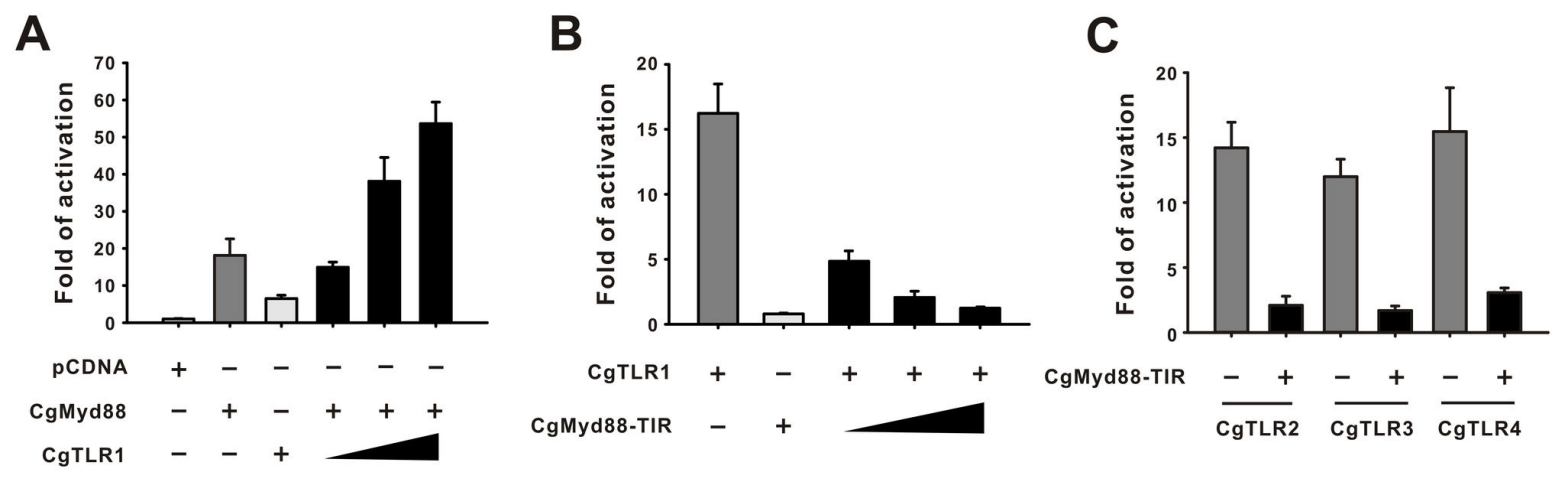
The NF-κB activity triggered by CgTLR-CgMyD88 linked pathway. (A) CgMyD88 induced NF-κB activation is CgTLR1-dependent. HEK293 cells were transiently transfected with combination of 100 ng CgTLR1 and 100 ng CgMyD88 expression vectors with 20 ng NF-κB-responsive reporter vector and 20 ng pRL-TK vector per well in 24 well plates. The triangle increasing symbol indicated that dose of CgTLR1 vector is transfected from 100 ng to 500 ng per well. (B) CgMyD88-TIR blocked the CgTLR1-triggered activation of NF-κB. HEK293 cells were transiently transfected with combination of 100 ng CgTLR1 and 100 ng deletion mutant CgMyD88-TIR expression vectors with 20 ng NF-κB-responsive reporter vector and 20 ng pRL-TK vector per well in 24 well plates. The triangle increasing symbol indicated that dose of CgMyD88-TIR vector is transfected from 100 to 500 ng per well. (C) CgMyD88-TIR inhibited CgTLR2-, CgTLR3-, CgTLR4-dependent NF-κB activation. HEK293 cells were transiently transfected with 100 ng of each CgTLR2, CgTLR3 and CgTLR4 expression vectors with 200 ng CgMyD88-TIR expression vector. Luciferase activities were tested at 24 h post transfection. Each column and error bar represents the mean±S.D. (n = 5).

To determine whether MyD88 is the conserved functional adaptor of TLR signaling, we also cloned the MyD88 homologue in oyster (CgMyd88) (NCBI No. KC700621). Similarly, CgMyD88 contained an N-terminal death domain, an intermediate domain and a TIR domain (Figure S1). Over-expression of CgMyD88 enhanced CgTLR1-dependent NF-κB activation when transfection of CgTLR1 expression plasmid increased from 100ng to 500ng per well (n = 5, p < 0.001; Figure 5A). The domain deleted vector CgMyD88-TIR not only failed to induce the NF-κB activation, but also strongly blocked CgTLR1-activated NF-κB. The block effect of CgMyD88-TIR was increased as transfection dose of CgMyD88-TIR vector increased (n = 5, p < 0.001; Figure 5B). Moreover, this mutant also inhibited the CgTLR2, CgTLR3 and CgTLR4 -dependent activation (Figure 5C), thus clearly confirming the universal function of MyD88 adaptors in TLR signaling.

### Expression of CgTLRs responding to acute immune challenges

**Figure 6:**
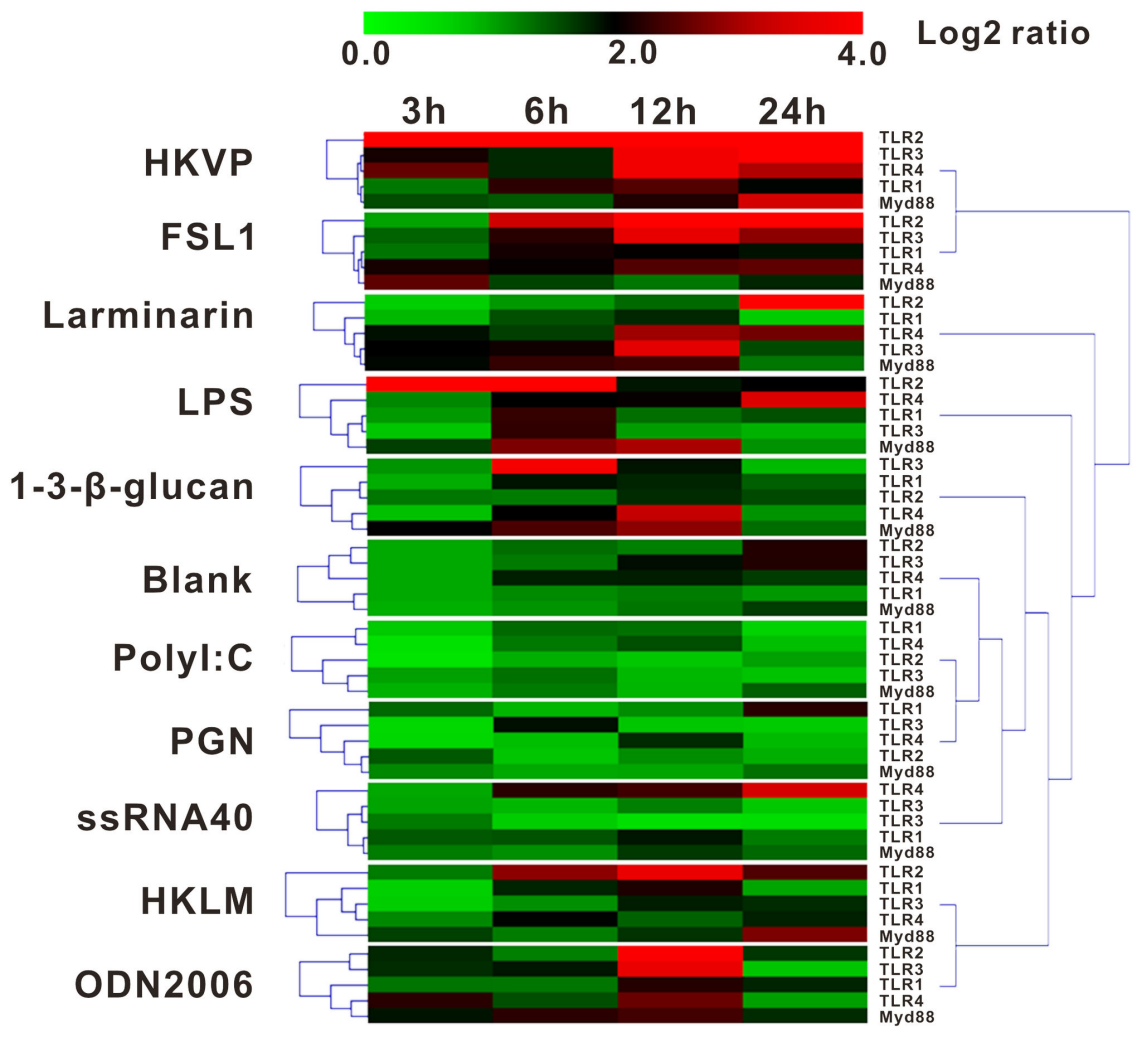
The mRNA expression patterns of CgTLRs and CgMyD88 in response to pathogenic ligands in hemocytes of *C* *. gigas* (n = 3). The mRNA expression was determined using real-time PCR in primary cultured hemocyte cells challenged with HKVP, HKLM and eight PAMPs post 3, 6, 12, and 24 h. All of the expression levels were normalized with geometric averaging of three reference genes EF1α, rpl, and GAPDH. The color code indicates the fold change of TLR mRNA after logarithm transformation, and the dendrograms were construed based on algorithm of hierarchical clustering.

The expression profile of CgTLRs response to immune challenges was analyzed using real-time RT-PCR in primary cultured hemocyte cells challenged with HKVP, HKLM and eight PAMPs. Generally, mRNA expression of CgTLRs and CgMyD88 increased in response to the PAMPs; specifically, HKVP and FLS1 elicited higher expressions of multiple CgTLRs and CgMyD88 than other PAMPs, while larminarin, LPS, ssRNA, glucan, ODN2006 and FSL1 only triggered moderate up-regulation of some CgTLR members, such as LPS activated CgTLR2, glucan for CgTLR3 and ssRNA for CgTLR4. However, PGN and Poly I:C had no significant effect on expression levels of CgTLRs and CgMyD88 when compared with the blank group (Figure 6). These expression profiles imply that CgTLRs are involved in host-defense in *C* *. gigas* , and show different magnitudes of response to specific PAMPs.

### Blocking peptide inhibits infection leading to the degranulation and activation of cytokines mRNA in hemocytes

**Figure 7:**
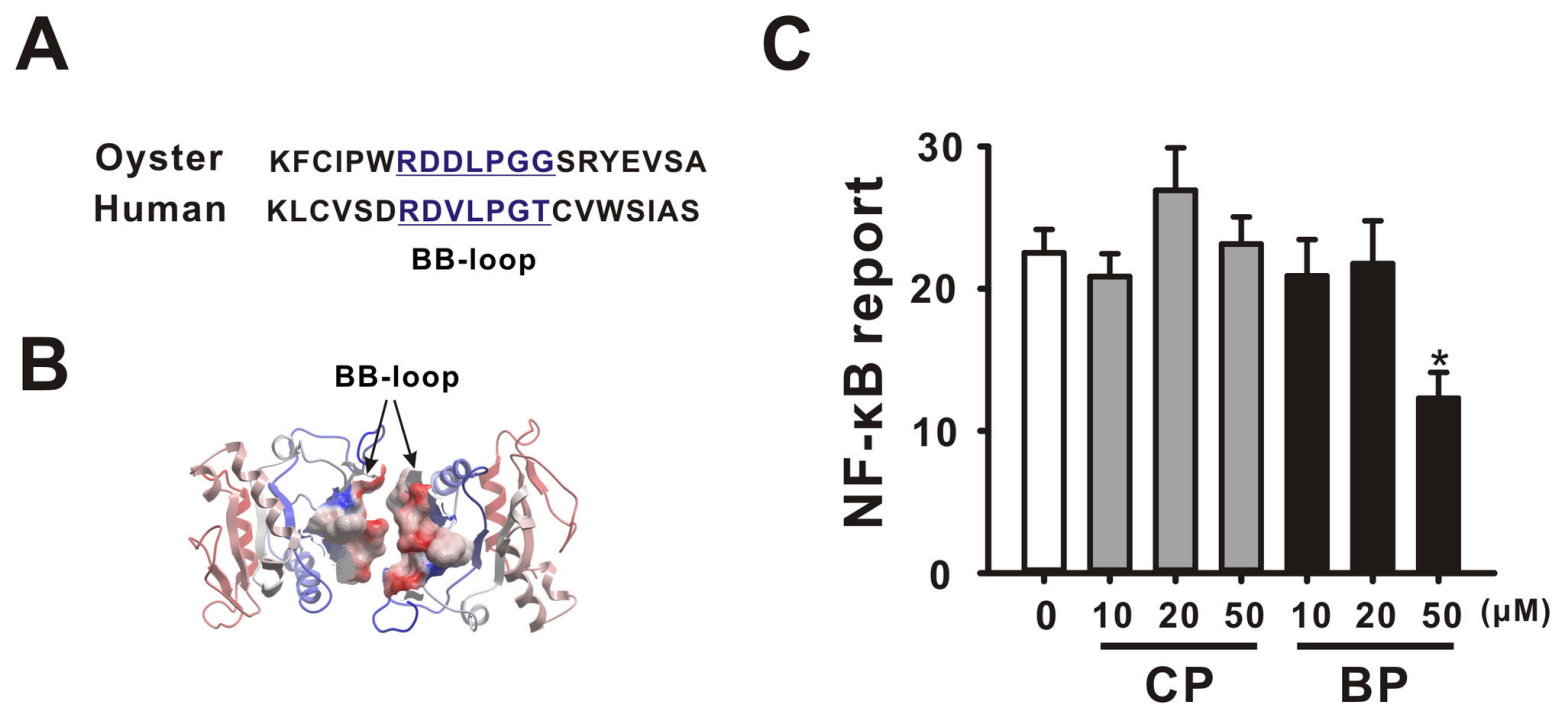
Blocking peptide targeted BB-loop of CgMyd88 inhibited TLR signaling. (A) Sequences of conserved motif of BB-loop from human and oyster. (B) 3D structure shows the location of a BB-loop on a TIR domain. (C) Inhibitory effect of blocking peptide on NF-κB activation. HEK293 cells were transiently co-transfected with 100ng CgTLR, 100 ng CgMyd88, 20 ng NF-κB-responsive reporter vector and 20 ng pRL-TK vector per well in 24 well plates under treatment of either CP or BP in a concentration of 10, 20 and 50 µM, respectively. Each column and error bar represents the mean±S.D. (n = 5). A start indicates p<0.05. CP: control peptide, BP: blocking peptide.

**Figure 8:**
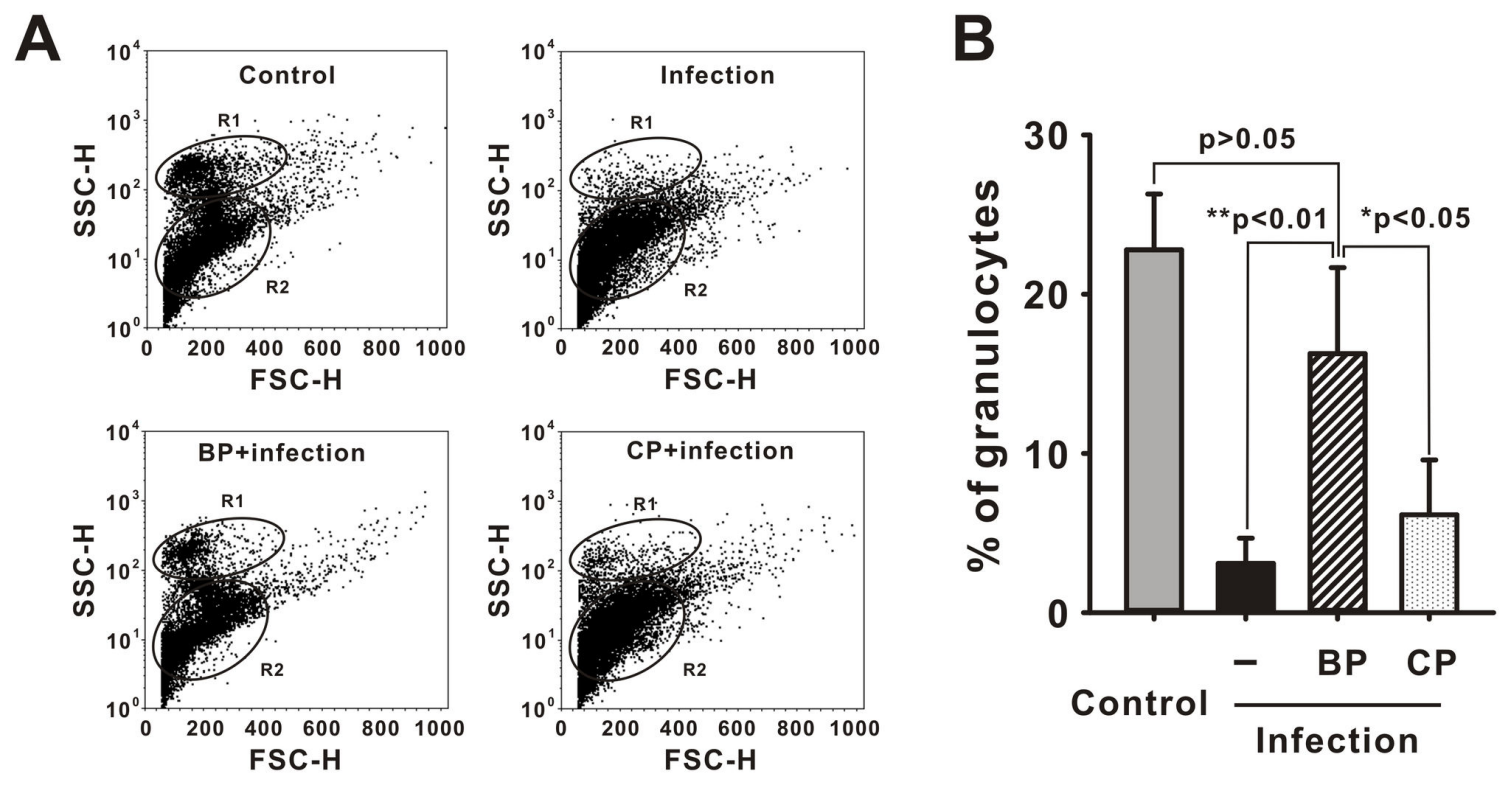
Blocking peptide (BP) inhibits degranulation caused by infection. (A) The granulocytes population in hemocytes under bacterial infection in presence of BP or CP. The hemocyte cells were plotted in a side scatter (SSC, Y-axis) versus forward scatter (FSC, X-axis) dot plot, and the different populations were analyzed and quantified using CellQuest. R1: granulocytes, R2: intermediate cells and hylinocytes. (B) The percentage of granulocytes population in hemocytes under treatments. Each column and error bar represents the mean±S.D. (n = 7).

The Blocking peptide exerted an inhibitory effect on NF-κB activation (by co-transfection of CgTLR and CgMyd88 at 50 µM; Figure 7C). Due to the strong response of CgTLRs and CgMyd88 expression to *V. parahaemolyticus*, we examined the role of Myd88-dependent TLR signaling in hemocytes infected with *V. parahaemolyticus*. Flow cytometry analysis showed that oyster hemocytes could be discriminated into two populations, R1 (granulocytes) and R2 (intermediate cells and hylinocytes), based on FSC and SSC. At 24 h post-infection, the R1 population decreased from 22.8% (non-infected control) to 3.5% (infection). The infection induced degranulation was inhibited by pre-injection with BP (16.3%), whereas injection with CP failed to block it and 6.1% of the R1 population showed evidence of degranulation which is comparable with that observed in the infected control group (Figure 8).

**Figure 9:**
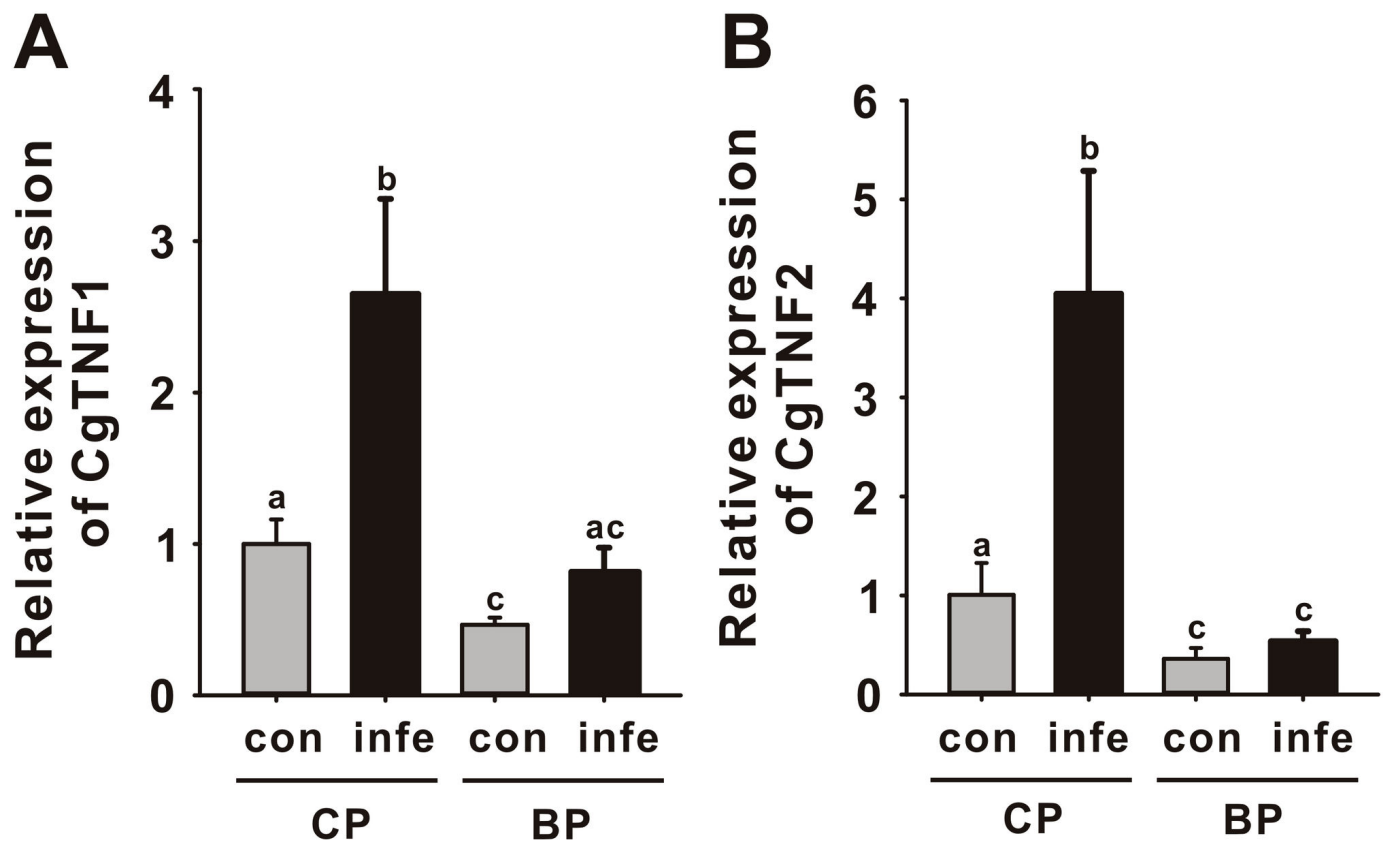
The mRNA expression levels of CgTNF1 (A) and CgTNF2 (B) in TLR signaling trigged by *V. parahaemolyticus* infection and controlled by blocking peptide. The mRNA expression was investigated by real-time PCR in primary cultured hemocyte cells infected with *V. parahaemolyticus* under treatment of either CP or BP. Each column and error bar represents the mean±S.D. (n = 7). Different letters indicate significant differences in expression between each treatment (p < 0.05). Con: control group, infe: infected group.

Inflammatory cytokine TNF is one of the conserved targets of TLR mediated NF-κB signaling in mammals. Therefore, we also examined whether expression level of CgTNF gene was subject to TLR signaling. Real-time PCR showed that *V. parahaemolyticus* infection can induce 2.7 and 4.1-fold increases in CgTNF1 and CgTNF2 in CP groups, respectively (n = 7, p < 0.05; Figure 9). Additional to blocking infection-induced activation of CgTNF1 and CgTNF2, the TLR inhibitor also inhibited the 2.2- and 2.8-fold increases in CgTNF1 and CgTNF2 mRNA when compared to the control group, implying an essential role for TLR signaling in activation of hemocytes.

## Discussion

The innate immune system is evolutionally conserved across vertebrate and invertebrate species, providing the first-line of defense by which a host organism can protect itself from pathogen infection [24-26]. However, the presence of huge structural and functional divergence between mammalian TLRs and insect Tolls makes TLR-mediated innate immune response an ongoing area of controversy [11]. To address this issue, we studied the role of TLRs in the immune response of a mollusk species ( *C* *. gigas* ; which is a member of the lophotrocozoans, a sister group of ecdysozoans), to provide insights into origin and emergence of TLR-mediated innate immune.

The four CgTLRs identified from *C* *. gigas* share conserved domain organization including two prominent functional domains: the tandem LRRs flanking LRR-N/CT for PAMP binding or recognition, and the intracellular TIR domain for signal transduction. The numbers of LRR of CgTLR1 to CgTLR4 range from 6 to 14. In comparison, their homolog in more ancient animals such as hydra *H* *. magnipapillata* ) and coral *A* *. millepora* does not bear LRR motifs [11], suggesting that LRR domain duplication or expansion may only occur in bilateral TLR lineage, followed by emergence of an ability for PAMP recognition. Notably, all CgTLRs show similarity to mammalian TLRs, as evidenced by their belonging to sccTLR but not mccTLR. Such structural organization is also found in other mollusk TLR families; most members possess sccTLR-type structures but minorities are mccTLRs, e.g. only three out of 23 TLRs from *Mytilusgalloprovincialis* , one out of 7 TLRs from *Euprymnascolopes* and four out of 70 TLRs from *Pinctada* *fucata* are mccTLRs [17,27]. All of these findings challenge previous assumptions that the distribution of sccTLR and mccTLR have corresponding relationships with deuterostomes and protostomes; most sccTLRs are found in deuterostomes, while most mccTLRs are found in protostomes such as insects, nematodes and mollusca [15-17,27]. Moreover, recent genomic analysis showed the co-existence of two TLR types in the ancient invertebrate deuterostome, *S. purpuratus* and *B* *. lanceolatum* [28], which strongly implies that both sccTLR and mccTLR have already presented in the bilateria ancestor, but have independently evolved and preferentially expanded, either respectively, or in individual species after deuterostome-protostome divergence.

The most striking difference between mammal TLRs and Drosophila Tolls is their ability to directly recognize their ligands. To address this, we examined the response of each CgTLR to known TLR ligands and PAMPs in mammal cell lines. Our results show that transfection with CgTLRs constitutively activates NF-κB reporter in a dose-dependent manner, suggesting an ancient and conserved link between TLRs and NF-κB signaling in mollusks. However, there was no evidence of a response when TLR ligands and PAMPs were used to stimulate CgTLR transfected cells. The lack of PAMPs recognition was consistent with previous reports for amphioxus and Drosophila [6,29]. In contrast, Sasaki et al. demonstrated that ascidian *Cionaintestinalis* TLRs show low level responses to high concentrations of PAMPs [30], however, such a response is too weak to distinguish from *C* *. intestinalis* TLR’s authentic ability and the background response of cell line. However, these evidences suggest that specific PAMP recognition of TLR might appear during evolution of vertebrates. It may also be possible that endogenous ligand was required for activation of CgTLRs (e.g. Spätzle in Drosophila [31,32]), however, further study would be required to address this.

In mammals, recognition patterns of TLRs correlate to some extent with their cellular localizations. Human TLR1, TLR2, TLR4, TLR5, and TLR6 locate on the plasma membranes, recognizing extracellular microbial pathogenic molecules, like lipopeptides (TLR1, TLR2 and TLR6), LPS (TLR4) and Flagellin (TLR5), whereas TLR3, TLR7, TLR8, and TLR9 are expressed on endosomes and involved in the recognition of nucleic-acid-like structures, such as dsRNA (TLR3), ssRNA (TLR7 and TLR8) and CpG-containing DNA (TLR9) [33]. The four CgTLRs have comparable subcellular localization patterns and are all present on membranes including plasma and late endosome (mainly on the latter), which is similar to ascidian Ci-TLR1 and Ci-TLR2 [30]. The lack of specific PAMPs recognition and specific cellular localizations may reflect a feature of the TLR ancestor, with specification of subcellular localization, along with PAMPs recognition, not emerging until the appearance of the vertebrate lineage.

From insects to mammals, Myd88 can act as a conserved adaptor to mediate canonical TLR dependent NF-κB activation [34-36]. Generally, myd88 protein contains a C-terminal TIR domain, which allows for interaction with TLRs, and an N-terminal death domain that is required for interaction with and recruitment of IRAK kinase [34]. To determine whether oysters share a conserved TLR signaling pathway, we cloned one Myd88 in oyster which shares similar domain organization with its human homolog. Our results showed that both the deletion mutant of myd88 and the blocking peptide (BP) that targets the BB loop in TIR domain of CgMyd88 can effectively inhibit TLR activated NF-κB reporter. This strongly suggests that MyD88 is a functional adaptor of TLRs signaling in oyster, and that they may have coevolved and remained conserved through evolution.

Given our evidence that the TLR- MyD88 signaling pathway is conserved in oyster, we next determined whether it is responsible for an innate immune response *in vivo*. The innate immune system is the first and only line of defense against pathogen infection in invertebrates due to the lack of adaptive immunity. The expression profile of CgTLRs and CgMyD88 in response to a wide range of PAMPs and bacteria were investigated in hemocytes, since they have been shown to be the key immune cells involved in protection of the host from infection, via phagocytosis and the release of antibacterial peptide or others [37]. Our results showed that multi-PAMPs can activate CgTLR and Myd88 transcription in oyster hemocyte, suggesting their involvement in pathogen infection of CgTLR signaling. In particular, HKVP and FLS1 induced the highest response highlighting the important role of TLR signaling in combating with gram-negative bacteria. Interestingly, this is comparable with TLRs in Drosophila, where TLRs contribute to host immune defenses and control resistance to gram-positive bacterial and fungal infections [38]. Thus, it is assumed that TLR-mediated innate immunity shared a bilateral ancestor, and then diversified in its form or evolved independently in different lineages to cope with different environments.

In hemocytes, granulocytes are considered as key active scavengers in eliminating invaders through the strategies of ingestion and release of anti-microbial proteins such as granule proteins. They can be recruited via appropriate signals from infection, traveling from blood or lymph stream into infected sites, where they initiate inflammatory responses and finally sacrifice for clearing of pathogens. We observed significant decrease of granulocytes under *V. parahaemolyticus* infection, indicating its link to the release of granular content for eliminating invading pathogens. Interestingly, infection causing de-granulation in granules can be inhibited by the blocking peptide of TLR signaling, revealing that TLR signaling can initiate and control the process of cell immune function in oyster.

Our further study also demonstrated that two TNFs (CgTNF1 and CgTNF2), the representative targets of TLR-NF-κB pathway in mammals, were up-regulated in hemocytes infected with *V. parahaemolyticus*. Further, their expression could be effectively suppressed by BP administration *in vivo*, strongly supporting evidence for a crucial and conserved role of TLR signaling in triggering inflammatory responses of oyster. This evidence supports suggestions that MyD88-dependent TLRs signaling is responsible for innate immune activation in hemocytes of oyster.

In conclusion, functional analysis of TLRs in a lophotrocozoan provides new insights to better understand the origin of TLR mediated innate immunity. The lack of ligand recognition and non-specificity of subcellular localization emphasized the primitive or ancient characteristics of TLRs even though its members in *C* *. gigas* have a considerable diversification in LRR organization. Moreover, our study provided compelling evidence that TLR signaling plays an essential and fundamental role in hemocyte activation and TNF induction caused by bacterial infection, revealing that TLR-mediated innate immunity shared an ancient origin at least in bilateria phylum.

## Supporting Information

Figure S1 **Nucleotide sequence and deduced amino acid sequence of TLRs and Myd88 from *C* *. gigas* .** The start codon (ATG) and the stop codon (TGA) are in bold. The predicted signal peptide is in italic type and in bold. The predicted leucine-rich repeats (LRR) are in boxes. The leucine rich repeat C-terminal domain (LRRCT) is underlined. The transmembrane segment is double underlined. The Toll-interleukin 1-resistance (TIR) domain is in shadow.
(PDF)

Table S1 **Sequences of primers used in this study.** “F” indicated forward primer, “R” indicated reverse primer.
(DOCX)

Table S2 **Sequences used for MDS analysis.**
(DOCX)
