# Supplementary material for: Characteristic and Functional Analysis of Toll-like Receptors (TLRs) in the lophotrocozoan, Crassostrea gigas, Reveals Ancient Origin of TLR-Mediated Innate Immunity
Source: PLoS One. 2013 Oct 1;8(10):e76464. doi: 10.1371/journal.pone.0076464 (PMC3788107; doi:10.1371/journal.pone.0076464)
Supplement: Figure S1 — Nucleotide sequence and deduced amino acid sequence of TLRs and Myd88 from C . gigas . The start codon (ATG) and the stop codon (TGA) are in bold. The predicted signal peptide is in italic type and in bold. The predicted leucine-rich repeats (LRR) are in boxes. The leucine rich repeat C-terminal domain (LRRCT) is underlined. The transmembrane segment is double underlined. The Toll-interleukin 1-resistance (TIR) domain is in shadow. (PDF) [file pone.0076464.s001.pdf]

TLR1

1 CGTGATATTGTCATGAAGAGATCGTCTACTTTTTCCCCATGATACATTACAAAT 54

55 TCAGGAAAATGTGACGAACAGCG **ATG** GAA GTG AAA ATA ACA GTT TTG TGG TCT TTT ATC CTT CTG 119

1 *M E V K I T V L W S F I L L* 14

120 CAA CTT GTT GTT TCT GAA CGA TGT GGG GAT GGA TAT TGC AAG TGT TCG AAG GCT CGA AAA 179

15 *Q L V V S* E R C G D G Y C K C S K A R K 34

180 GTT GCC ACG TGT ATG TCT AGA AGG AGG CAA TTA AAT TAC TTT CCT TCA TTG CCC ACT TAC 239

35 V A T C M S R R R Q L N Y F P S L P T Y 54

240 GTA AAA CAG CTC AGA TAC AGT GGC AAC TAT CTG CCC CAC CTT ACT CGA GAC TTA ATG ATT 299

55 V K Q L R Y S G N Y L P H L T R D L M I 74

300 AAT TTG ACC AGG GTC AAT CTA GAA CAA CTC TAT TTA GAA GAT AAT GTG ATG TTC AAA GTT 359

75 N L T R V N L E Q L Y L E D N V M F K V 94

360 GAT CCA GAT GCC TTT GAG AAT TTG ACA ACT CTG GTT AAA CTT CAA ATT TCT CAT GAC AAG 419

95 D P D A F E N L T T L V K L Q I S H D K 114

420 ATA CTC AAT GCC AGT ATT GTT GCA CAA GTT CTC CAT AAT GTG AGT CGT TCT GTT AAA AAG 479

115 I L N A S I V A Q V L H N V S R S V K K 134

480 TTA GTA TTA AAC CAC AAC AAA TGG AAT TCG GTA CCG GAC AAC ATC TTT GAT GGT CTA AAA 539

135 L V L N H N K W N S V P D N I F D G L K 154

540 GAT TCA AAC ATT CAT TTC ATC GAC TTG TCT TTT AAC AAC TTG CAT TCA TTC AAC GGT TTG 599

155 D S N I H F I D L S F N N L H S F N G L 174

600 AGG ATT TTT CGA AAT ATT CCA AAT CTT AAA TAC TTG ATT TTG GAT GGA AAC CCC ATA TTG 659

175 R I F R N I P N L K Y L I L D G N P I L 194

660 GAC AGT GAT ACA GAT TTC CAT AAC ATG GAA TAT ATA TTT AAA TTA AGT TTA CAA TAT ACG 719

195 D S D T D F H N M E Y I F K L S L Q Y T 214

720 TTC ATA CAA AAC GTT CCC ACA TTT TGT GAT GAC AAT GGG GAA AGT CTT CTG CCC CGC ATG 779

215 F I Q N V P T F C D D N G E S L L P R M 234

780 CGA TCT CTT AAT TTG GAC CAT TCT TCA TTA CGA TCC ACC TCT CGA AAC GAT TTT ATT TGT 839

235 R S L N L D H S S L R S T S R N D F I C 254

840 CTG CCC GAT CTT TGG AAA TTG TCC CTT TCA TTT ACA GGC ATC ACC GTG ATA CAA AAT GAC 902

255 L P D L W K L S L S F T G I T V I Q N D 274

900 ACG TTT TTG GAT ATG CCA GTA CTT CAG ACT CTT ATG TTA CAA AAT GTG GTG AGG CTC AGA 959

275 T F L D M P V L Q T L M L Q N V V R L R 294

960 CGC TTA GAA TAT TTC GCG TTT CGC TCT GCT TCT CTT CAA TCG TTC AAA TTT GGG TCG TCA 1019

295 R L E Y F A F R S A S L Q S F K F G S S 314

1020 AAA TTT AAA TTT GAC AAC AAA GGT CGA TAC AAT AAT GAT TTG TTT AAG TTT GCG CCA AAC 1082

315 K F K F D N K G R Y N N D L F K F A P N 334

1080 ATA ACA AAT ATT GAG CTA TTC GAC AAT CAG ATT TCT GAT GGA TCT ACT CTG AAA ACA CTT 1139

335 I T N I E L F D N Q I S D G S T L K T L 354

1140 TTG TGG AAC TTG ATT AAG TTG CAG AAG TTG AAT CTC CAG GGA TGT GGG ATA AAT TAT CTT 1199

355 L W N L I K L Q K L N L Q G C G I N Y L 374

1200 GCA CGT GGA ACA TTC GAT AGA ATG CCA GAT CTT CGA ACA ATT ATC TTA AAA GGC AAC AGC 1262

375 A R G T F D R M P D L R T I I L K G N S 394

1260 TTG TAC GGG TGG GAT CCC GAC ATG TTT AAC AAA CTT TTC AAT TTA AGA GCC CTG TAT TTA 1319

395 L Y G W D P D M F N K L F N L R A L Y L 414

1320 TCA GGG AAC AGT GTT GCC GTT GTT AGC AGG ACT TCT CTT GCG TTT ATC GGC AAA ATT AAT 1379

415 S G N S V A V V S R T S L A F I G K I N 434

1380 CTC AAA TTT ATT GAT TTA GCA GAC AAT CCG TTT GCA TGT ACC TGT CAA CAG CTT TGG TTC 1439

435 L K F I D L A D N P F A C T C Q Q L W F 454

1440 CGT GAC TGG TTA AAA ACA GCC AAA AAT ATT ACA GTT GCC TTT TAT CCG AAG AGA TAT GTC 1499

455 R D W L K T A K N I T V A F Y P K R Y V 474

1500 TGT AGA TCT CCC CCT AAA TGG GAC AAT ACT TTG GTA GCT TTA TTC AAT TAC ACC GAA GAA 1559

475 C R S P P K W D N T L V A L F N Y T E E 494

1560 GAT TGT CGG GAG AAA AAT CCT TGG ATT TTG ATA GGA AGT GTT CTA GGA TCG GTT GTT TTT 1619

495 D C R E K N P W I L I G S V L G S V V F 514

1620 GTG TGC ATG GTT GTC GTC ATC GTC ATA TAC ACA CAT CTT CCG ACC GTC AGA AAT ATC ATC 1679

515 V C M V V V I V I Y T H L P T V R N I I 534

1680 TAT TTG ATT CGT TTA CGC AGG AAA GGC TAC GTG AGA CTG GTG AAC TCC GAG GAG TAC ATG 1739

535 Y L I R L R R K G Y V R L V N S E E Y M 554

1740 TTT GAC TGT TAT GTC GTT AGC TGT GAA ACT GAT GAA CAA TGG GTG TTC CAA ACT CTG TCT 1799

555 F D C Y V V S C E T D E Q W V F Q T L S 574

1800 TCT ACT TTA GAG GTT AAA CAT TCT TAT CGT TTG TGT ATA CCT ACA AGA GAT TTT GAC ATC 1859

575 S T L E V K H S Y R L C I P T R D F D I 594

1860 GGT GCA AGT ATA GCT GAT CAG ATT GAA GAA AAA ATG AGG GAA AGT AAG AAA ATC ATC ATT 1919

595 G A S I A D Q I E E K M R E S K K I I I 614

1920 GTG ATG TCA AAC GAT TTT GCT CAG GAC GAA TGG TGT CAG TTT CAG CTA GAG AAA GCA CAG 1979

615 V M S N D F A Q D E W C Q F Q L E K A Q 634

1980 GAG CGG ATC CGA AAC CAA GGA GAA GAA GCT GTT GTG TCA ATC ATG CTG CAC GAT ATT GAT 2042

635 E R I R N Q G E E A V V S I M L H D I D 654

2040 CAT AAA CAT ATG ACA TCA ACC ATC AAT AAT TTG TTA CGT AAA AGT TCG TAT GCT ACT TGG 2099

655 H K H M T S T I N N L L R K S S Y A T W 674

2100 GTG AAA GGT AAA ATT GTC AGT AAG CTG TTC TGG GAC ATT GTT GTT GCA GCT ATA GAA AAA 2159

675 V K G K I V S K L F W D I V V A A I E K 694

2160 CCT TTT GGT AAT CCA CCA ATT GCT ATT **TGA** CGAAAGTCGGTGCCGATCACCGGGTCCTCATTCAACACA 2228

695 P F G N P P I A I * 703

2229 GAAGAGACTTACTGAAATTGTCACTTACAATTATTTGTTATGACATATTTTAATTATAAAAAGGACAATGGAACATTCT 2307

2308 CAGTATTAACTGTAGCAGTATTGTTGTGAAAATGAAATTCATTTTTGTAGAATATCTTATTGCATAATAATTAATTCAT 2385

2386 GAGTACTCTGAAA**AATAAA**AAAAATAACCCAAACATAAAAAAAAAGAAAAAAAAAAAA 2445

TLR2

1 TTGACACTGGAATGGACTGAAGGAGTAGAAAAATATTTGTGTTTTGAGAACATCGCC **ATG** 60

1 *M* 1

61 ACC AGA GCA CAG ATT TTG TTC CTT GCT TTT GTG TTG GTG TAC GAA AGT GTA CAT CCA AAG 120

2 *T R A Q I L F L A F V L V Y E S V H P* K 21

121 CCA TGT GAG CGA AAT GAA AAA CCG CCC GAT GGG TGT ACA TGT TCA AAA TAT GAA GGT AAA 180

22 P C E R N E K P P D G C T C S K Y E G K 41

181 CAG TAT TAC GTG ACA ATT AAG TGC GTG AAT AAA GGA GAA AGT CCA CGA AGT GAT ACT ATT 240

42 Q Y Y V T I K C V N K G E S P R S D T I 61

241 CCC TCG ATG CCA AAC AAT ACT TAC CAG TTA ATC ATT CAA GGA TTT CGA TTC ATA AAT GTA 300

62 P S M P N N T Y Q L I I Q G F R F I N V 81

301 ACT AAG AAA ACG TTC GGT AAT TTG AGG AGA CTT TCT TCC CTG CAT GTA TTA AAT TTA CTG 360

82 T K K T F G N L R R L S S L H V L N L L 101

361 GAT AAT AAC ATC ATC ACC ATT TCC CAT GAT GCT TTA TCC GAA TTG AAA CAT TTA AAG GAA 420

102 D N N I I T I S H D A L S E L K H L K E 121

421 TTG GAG ATC AGC TGG GAA ATC CAA GTG AAT AGG AGA GAA ATA TCA GAG ATG CTT TCT TAC 480

122 L E I S W E I Q V N R R E I S E M L S Y 141

481 ATC ACT CGC AAC ATT ACG TTT ATC AAA TTC TCC CAT AAT GCC TGG GAC CAA CCG CCT GAT 540

142 I T R N I T F I K F S H N A W D Q P P D 161

541 TTT GCT GGA CTG TGG AAT GCA ACA CTT CGA AAT TTA ACC CTG TCC TAT AAT CAT TTC ACT 600

162 F A G L W N A T L R N L T L S Y N H F T 181

601 GCA TTG GAA GGT TCT AAC TAT TCC AGT TTA AAT CAA CTT CGC AAA TTA GAC GTA TCA TAC 660

182 A L E G S N Y S S L N Q L R K L D V S Y 201

661 AAT GGA ATT ACG GAA AAT GGG AAT AAT TTT ACT GGA CTT GAA AAT ATC AAC GAA TTG GTT 720

202 N G I T E N G N N F T G L E N I N E L V 221

721 TTG GAT GGA AAC TGG TTT AAA GAA TTT CCA AAG TTT TGT GAC TAT AAA TTT TCT AAT TTG 780

222 L D G N W F K E F P K F C D Y K F S N L 241

781 TCA ACA ATC TCG TTT AAG AAT AAT AAA TTA ACT GAA TTT AGA TCA ACA TAT TTC CAG TGT 840

242 S T I S F K N N K L T E F R S T Y F Q C 261

841 TTA ACA AAT TTG CAA GCC TTG AAT TTA AAT GGG CAT GCA ATA AGA AAG TTG TAT AAC AAT 900

262 L T N L Q A L N L N G H A I R K L Y N N 281

901 ACA TTT ACC AAT CTG ACG TCA TTG CGT CAA CTT TAC ATT AAG CGG TTG GCT GGA CAA CTT 960

282 T F T N L T S L R Q L Y I K R L A G Q L 301

961 TCC CAC ATT GAA CCA GAA GCG TTT AAA AGC AAC TCC CTT CAA GAA CTC AGG TTT TCT TAC 1020

302 S H I E P E A F K S N S L Q E L R F S Y 321

1021 AAT GGA TTT TTC TTT TCC GAT TTC CCC AAA GAA ACC CTC GTT ATG TTC AAA TAC TGC CCA 1080

322 N G F F F S D F P K E T L V M F K Y C P 341

1081 AAA TTG ACC TTA CTA GAT ATT TCA GCA AAC CAT CTT AAA TTA AAG GAA AAC GAA CTC ACA 1140

342 K L T L L D I S A N H L K L K E N E L T 361

1141 AAG ATG ATA AAA AAC TTG AAA AAT GTT ACA ACA CTT ATT ATT AGA AAT ACA GGA TTA CAC 1200

362 K M I K N L K N V T T L I I R N T G L H 381

1201 TAC CTT CCG AGA AAT TTG ACA CGT CAT CTG CCG ATG TTA AGG AAA CTC GAC GCT TCG GAC 1260

382 Y L P R N L T R H L P M L R K L D A S D 401

1261 AAC TAT TTA AAC GGA TCA TGG GAC GGG AAT TCT GTA TTT GGA AAC GTC CCA TCT TTG CAA 1320

402 N Y L N G S W D G N S V F G N V P S L Q 421

1321 TAT CTG GAT TTG TCG AAA AAC TAC ATT GAA AGA ATC ACT GAA CAT AAT TTT CCA CTA ACT 1380

422 Y L D L S K N Y I E R I T E H N F P L T 441

1381 CTA TTG AAT GGA TTA GAT GAG GGC GGG TTG GAT CTT AGC TAT AAT AAA CTC TCT TGC AAT 1440

442 L L N G L D E G G L D L S Y N K L S C N 461

1441 TGC GAT GAC GCG CTA TGG TTT TAC AAT TGG ATG CAT GCC AAT GAG AAA AAG TTA TCA CAT 1500

462 C D D A L W F Y N W M H A N E K K L S H 481

1501 GTT GAA AAT ACA ACA TGT CGA CTC AAT GTT CCT CTT GAT GCG AGC ACG AGA AAT TTG TTC 1560

482 V E N T T C R L N V P L D A S T R N L F 501

1561 TAT CTC ACC GAA AAA GAA CTG TGT CCA ATG AAT CCT GCT GTG TTC ATT GCT ATC GTA AGT 1620

502 Y L T E K E L C P M N P A V F I A I V S 521
1621 TCA GGC ACC GCC TTA TTG GTG ATT TTT ATC ACG TTT GCA GCT TTA TAC AAG CTT AGA TGG 1680
522 S G T A L L V I F I T F A A L Y K L R W 541
1681 CAT ATA CGT CAC TGG CTT TAC GTC ATA AAA TAT AAA AAC AAA GGG TAC GAA ATC ATA CCG 1740
542 H I R H W L Y V I K Y K N K G Y E I I P 561
1741 GAC GAC CCC GAT TTT AAA TAT GAC GTA TTT TTA GTT TAT GCC GAT GAG GAT ACT AAG TTT 1800
562 D D P D F K Y D V F L V Y A D E D T K F 581
1801 ATT TTT GAC ATT GTT GTG CCA TAT TTG GAA GGA AAA GGC TGC AGT TTG TGT GTG AGA TGT 1860
582 I F D I V V P Y L E G K G C S L C V R C 601
1861 CGG GAC TTT GAG ATA GGG AAG TTG TAT TGT GAC AAC ATA GTT GAC AAC ATG AAT CTC AGC 1920
602 R D F E I G K L Y C D N I V D N M N L S 621
1921 AGA CGT ATT CTA TTG ATT CTG TCT AAC AAT TTT GCC AAA AGC AAA TGG TGT GAA TTT CAA 1980
622 R R I L L I L S N N F A K S K W C E F Q 641
1981 ATG AAT TTT GCA TAT AAC AGG TGC TTG GAT GAA AAA ATA AAC AAC ATA ATT GTG GCA GTT 2040
642 M N F A Y N R C L D E K I N N I I V A V 661
2041 TGG GAA GAA ATA AGC TAC AAG TAT TTA TCG AAC ACT CTC AAA GTC TTG CTG ACG TCA TAC 2100
662 W E E I S Y K Y L S N T L K V L L T S Y 681
2101 GAC TAT GCT CTT TGG TCG AAG TCC GAT GCG ACT GGA CGA AGT TTG TTT TGG GGG AAA ATT 2160
682 D Y A L W S K S D A T G R S L F W G K I 701
2161 TTG CGG AAA TTA CAA TTT CAA TCT GGC GAC ACC GAG TGT GCT AAT GGT CTG CTC GGT GTT 2220
702 L R K L Q F Q S G D T E C A N G L L G V 721
2221 CTC CAA **TAG** GAACATGGAACAAAACCTATTTACAAATGCTTTCAAAAGTCTGCGTAATTCTCTGTCCTAAAACATC 2296
722 L Q * 723
2297 TGTGATTACAAACTGTTTGTTGTACCCATTAGTATCTCATGCACACACCTATCATTTAATTTCCAGAGAGAGCGATCAA 2375
2376 GATGTGATTTTTAAAAATGGCAAAATCTGTCATTATTTTTCTAGCGCATATGTAACATAGTCATGTCAAACTATTTTTT 2453
2454 TTA**AATAAA**TCCTACCCAATACCAAAAAAAAAGAAAAAAAAAAAAA 2500

TLR3

1 GTTTGACTCATTGCAACACC 20
21 GCACTAAAATGGAGCAATTTTAATTGCGTTAAACAGTTTCCAAGGACTCGATCTCAAGCACCGCCAGCACGTTTATTAC 99
100 CTGCAGTGCTGCTGATGATATAAATTATCCGGTTACGGACCACAAAATAAAGATCGAGAGCACATATTGACAAGATTTG 178
179 TGATTCTAGTACATCAAAA **ATG** AAG ATG CAT ACT ACT CCA GTA CTT CTA GTT GGC ATA TGC CTG 242
1 *M K M H T T P V L L V G I C L* 15
243 TTG CAA GCT GGT GTT GCA TGG GCA GAT ACA TGT CCC GCG TAC TGC TTT TGC AAT AAA TTA 302
16 *L Q A G V A W A* D T C P A Y C F C N K L 35
303 CTC ACG TCG GTG AAT TGC GAA GGC AAA AGG TTG ATG AAA ATA CCT ACA GAC CTG CCA AAA 362
36 L T S V N C E G K R L M K I P T D L P K 55
363 ACG GTC GAA AAA TTG TAT CTG CAG CAC AAC GAA ATT GCT GAT CTA GAG CCG AAC TCT CTC 422
56 T V E K L Y L Q H N E I A D L E P N S L 75
423 TGT GGG CTT TCC GAA TTG CAG GAA CTT TAT CTA CAG AAT AAC AAA CTT TCC TTT ATA AAG 482
76 C G L S E L Q E L Y L Q N N K L S F I K 95
483 AGT TTG ACA TTT ACC GGA ACA TGT GTA CCG AAT TTA AAA GTG ATA CGA CTT GAC AAT AAT 542
96 S L T F T G T C V P N L K V I R L D N N 115
543 CGC ATC AGT TCG TTG GAA GAA AAT GCA TTT TTC AAC ATG ACA AAC CTC AAT ATT ACC TAT 602

116 R I S S L E E N A F F N M T N L N I T Y 135

603 TTC ACT AAT AAC GTG ATC ACA CAT ATT AAT CCG CGT TCA TTT GTT GAA TGT TTT AAG ATG 662

136 F T N N V I T H I N P R S F V E C F K M 155

663 TCT TTC TTG CAT TTA GGA CAA AAC TAT TTA GAT CAC ATT CCA GCG ATA TCA TTT CTA ACT 722

156 S F L H L G Q N Y L D H I P A I S F L T 175

723 GGA CTA CAG CAG CTC AGC ATA CAA GGA AAC AAA GTA AAG AAC GCA ACA TTT CCA ACT AGC 782

176 G L Q Q L S I Q G N K V K N A T F P T S 195

783 TAT GAA AAC TGC ACA CTG CTG TCA ACA ATT GGT CTT AGC ACA AAT TTC ATA GAA AAT CTC 842

196 Y E N C T L L S T I G L S T N F I E N L 215

843 ACT AAG GAA ACT TTT CAG AGT CTG CAA AAT TGT CCC GTC AGA AAG TTG GAG TTA TCG AGA 902

216 T K E T F Q S L Q N C P V R K L E L S R 235

903 AAT AAA ATA ACT GAC ATT TCC AAG GAA GCC TTT CTG CCT TTA ACA AAA CTG GTG TCT CTT 962

236 N K I T D I S K E A F L P L T K L V S L 255

963 ACA ATC AGC CAA AAT CAT TTA ACG GCC CCT AAA CTT AAA ATT GGA TTA GAA GGT CTC AAA 1022

256 T I S Q N H L T A P K L K I G L E G L K 275

1023 TCC TCC TCG CTC AGC TCA CTT AAT ATA GCA AGA CTT CAA CTT GGA GGA CAA TTG CCC TCT 1082

276 S S S L S S L N I A R L Q L G G Q L P S 295

1083 TCC ACA TTT GCA CTT CTT AAT GGA ACA GTA CTG AAG CAA TTG CTT ATG AGT AAT AAC AAA 1142

296 S T F A L L N G T V L K Q L L M S N N K 315

1143 ATC AAT CAA CTT CCT AGT CGT GCA TTT GCC ACT TTA AAA AGG CTA GAG CAG ATC GAC TTG 1202

316 I N Q L P S R A F A T L K R L E Q I D L 335

1203 AAG GGC TGC AAG ATT CAG ACA ATT GCC AAC GAC ACT TTT GCA GGA CTC CAC TTT CTC ACC 1262

336 K G C K I Q T I A N D T F A G L H F L T 355

1263 AAT TTG AAC CTT GCA GAC AAC TAT TTG GAC AAA GTT CCC ACA AAT CTT CCA TCG TCG CTG 1322

356 N L N L A D N Y L D K V P T N L P S S L 375

1323 AAT ATA CTA TAT CTC AAT GGA AAT CAA ATC ATT GCT CTT GGT GAA AAT TCA TTC GTG AAT 1382

376 N I L Y L N G N Q I I A L G E N S F V N 395

1383 CTA GTA TCG TTG AAA AAC CTT TAC CTG GGT GCC AAC AAA ATT TCT GAG GTA AAC AAA TTG 1442

396 L V S L K N L Y L G A N K I S E V N K L 415

1443 GCT TTC AAT GGT TTG GTC AGC CTA CAA AAA CTG CAT TTG GTT TCC AAT AGT ATA AGT AGT 1502

416 A F N G L V S L Q K L H L V S N S I S S 435

1503 CTA GCC GCA GAA CTG TTT GCT CCA TTC GGA AGG CTG ATT TCC CTC GAA CTA AAT AAG AAC 1562

436 L A A E L F A P F G R L I S L E L N K N 455

1563 AAT TTG AAA ACA GTT CAA AAT TCA CCA GAC ATT TTC TCA TCA ATG ACG TCC TTG TTG TAT 1622

456 N L K T V Q N S P D I F S S M T S L L Y 475

1623 CTT TCA TTG GCT GAT AAC GGC TGC AGT ATA ATG CCA TTG TCG TCA TTC AAA CAC TTG CAA 1682

476 L S L A D N G C S I M P L S S F K H L Q 495

1683 TCA CTG AAA TAC TTG TTA CTT GAT GGT AAT AAT CTT GGA GAT CTA ATT GGA AGT GAC AAT 1742

496 S L K Y L L L D G N N L G D L I G S D N 515

1743 TCT GGG ACT TTG TTT GCT GGA TTA CAC AAA CTA GAA ACA CTT AGT CTT TCA AAG AAT TTC 1802

516 S G T L F A G L H K L E T L S L S K N F 535

1803 CTC CAC AAC CTA CCA ACA TCG ATG TTT AGA GAT CTT AGC TCC TTA AAA ACA TTA ACA ATG 1862

536 L H N L P T S M F R D L S S L K T L T M 555

1863 CAG GGC AAT AGA ATT TCT GGG TGG AAC AAT GCT TTA TTC AAA CAA ACT TCT GCG CTC AAA 1922

556 Q G N R I S G W N N A L F K Q T S A L K 575

1923 ACC CTA GAT CTT AGT GAC AAT TCT ATA TCG TTA GTG AAT TCA ACT TCC TTG GAA GAT CTT 1982

576 T L D L S D N S I S L V N S T S L E D L 595

1983 TCA CAA AAC CAA AAT TTT CAG ATG CTG AAT CTG AGC AAC AAT CCA TTT GCA TGC ACT TGC 2042

596 S Q N Q N F Q M L N L S N N P F A C T C 615

2043 GAC CTC CGA TGG TTC AGA GAT TGG GTA AAC CAA ACT AGA GTG AAT ATT GCA AAT GTA GAA 2102

616 D L R W F R D W V N Q T R V N I A N V E 635

2103 AAT TAT GTG TGT AAT TCT CCC GAT GCA TGG AAG GGG AAA CCT TTG TTA GTA TTT GAT CGG 2162

636 N Y V C N S P D A W K G K P L L V F D R 655

2163 ACA AAA ATA AAC TGT ATT TGG TTT AAC CTG TAT TTT GTA GTC GGG GTT AGT ACA GCC TCT 2222

656 T K I N C I W F N L Y F V V G V S T A S 675

2223 GGT ATC CTA GTG CTT GTC GTC TGC GTA ATT ATC TAC AAG AAG CGA TGG TGG ATT CTC TAC 2282

676 G I L V L V V C V I I Y K K R W W I L Y 695

2283 CGA TGT TAC CAA ATG CAA AAA TTG TGC ATG GGT TCG GAA AGA AGC AAA AAT GGT TAT CAG 2342

696 R C Y Q M Q K L C M G S E R S K N G Y Q 715

2343 CCA ATT AAC GGA TAT GTA TTT GAT GCT TAT ATA TCC AAT GCA GAT GAG GAC GAT AAA TGG 2402

716 P I N G Y V F D A Y I S N A D E D D K W 735

2403 GTT ATT GAA CAG CTA CTG CCA GAT ATC GAC TCT GGA GAA TTG ACT GAA GTC CAT GGA GAC 2462

736 V I E Q L L P D I D S G E L T E V H G D 755

2463 TTT AAG TTG TAT TTC AAA GCT CGT GAT GCA ATT CCC GGA AAA CGT GAC TTT TAC AAT ATC 2522

756 F K L Y F K A R D A I P G K R D F Y N I 775

2523 TTA GAA AAC ATT AAA GTA AGT AAA AAA GTC ATC ATC GTT TTG ACA GAA GAA TAC ATC TCC 2582

776 L E N I K V S K K V I I V L T E E Y I S 795

2583 AGT GAT CTA CAT CAA TTC GAA ATT GAT CTT GCT GTG AGC TTG AAA TAC AAG GGT GAA ATT 2642

796 S D L H Q F E I D L A V S L K Y K G E I 815

2643 GCT GAC ATT ATT GTC ATC AAT GTT GGT GGT GTT CCG TAT AAA CGC ATC CCA AGT TCA TTA 2702

816 A D I I V I N V G G V P Y K R I P S S L 835

2703 GAA TCG AAA ATT TCC AAA GAT GAA TTT TTG CTA TGG GAA AAT AAA GAC AGT GCC ATA AAG 2762

836 E S K I S K D E F L L W E N K D S A I K 855

2763 GTA TTC AAA GAA AGG CTC CAA AAG GAA CTA AGA CGT GAA ACG ATA ACT AAA CAT GTC GTT 2822

856 V F K E R L Q K E L R R E T I T K H V V 875

2823 GTA **TAA** TATATGATTGAAGGCTGTCATATAATTTTTTTTCAATTTCAGTTCCATTTGGGGTTTGCCGTGATGTTTA 2899

876 V * 876

2900 GATTTTATTAGGCTAATATATATAC**AATAAA**ATGCAATTTCTTTCGAAAAAAAAAAAAAAAAAAAAAAAAAAA 2972

TLR4

1 ACAATAACCACGCTACAGAACCAAGGAAACAGAAAGGACGACAGATT **ATG** AAG TAT TTA CGG 62

1 *M K Y L R* 5

63 ACG TTG ACA GTC TTT CTG TTG TTG GCC ATT CAT ACG TGT ACA CCA GAT GAA ATG GCT TCA 122

6 *T L T V F L L L A I H T C T P* D E M A S 25

123 GCG TCA CAC TGC CCG GAC TCC GTC TGT TTC TGT CGG GTT TTG GAC ACC GCA GAC TGC TCT 182

26 A S H C P D S V C F C R V L D T A D C S 45

183 TAC AGA AAT CTC AGC AAT ATA CCA CGT GGT CTC CCA GCC TCG ATT CGG TCT CTG GTT ATG 242

46 Y R N L S N I P R G L P A S I R S L V M 65

243 TCG GGG AAC CCA TTG TAT TCT CTT TCC GAT GAT TTC TTT CGT CCT GTA GAA AAC CTT ACC 302

66 S G N P L Y S L S D D F F R P V E N L T 85

303 TTG AAG AAT TTA GCT CTA GAT AAA TGT AAT ATT TTC AGA ATT TCT ACC TTT ACT CTA GAA 362

86 L K N L A L D K C N I F R I S T F T L E 105

363 CCA TTG AGA TCA CTA GAG ATG TTG GAT CTT TCC AGT AAT AAG TTA TAC AAC GCC ATG TTA 422

106 P L R S L E M L D L S S N K L Y N A M L 125

423 GAT AAA TGT ACT GAG AAA CTA GGG TAC TCG ACC CTT AAG GTT TTG AAC TTG TCG GGA AAT 482

126 D K C T E K L G Y S T L K V L N L S G N 145

483 TCG TTG TAT ACT TTA GAC GCT ATA TAT AAT TTT TTG GAA AAC AAA TGG TTT TCA AAT TTG 542

146 S L Y T L D A I Y N F L E N K W F S N L 165

543 ACA GAC CTT ATT ATG CAA CAA AGT CAG GTT AGA AAA GTG GAA ATG AAG AGT TTA AAA AAT 602

166 T D L I M Q Q S Q V R K V E M K S L K N 185

603 CTT CTT TTT CTG CAA ACA CTT GAT TTG AGC CAA AAC GCC ATA GAA CAA TTT TCT TCA TAT 662

186 L L F L Q T L D L S Q N A I E Q F S S Y 205

663 GGA TTG CCG ACT CTA AAA ACT TTG GAT CTG TCA TCG AAC TCC CTT CCA GAA TTC CCG AGG 722

206 G L P T L K T L D L S S N S L P E F P R 225

723 CCT TGC AAT GGT TTA AAT AAT CCT TTT TAT CCG AAA TTA GAG AAC CTA CTT CTC CGC AGA 782

226 P C N G L N N P F Y P K L E N L L L R R 245

783 AAT CGA ATC TCG GAG ACC TAT TTA CTC GAA GAT TAC GGA CAT TGC TTA ACA GCT TTG GTC 842

246 N R I S E T Y L L E D Y G H C L T A L V 265

843 AAA TTA GAT CTT TCT CTG AAT CCC ATT AAG ATT ATT CTG CCA TTT GCT TTT GTA TAT TTT 902

266 K L D L S L N P I K I I L P F A F V Y F 285

903 AAG GAT TTG ACA TAT CTA TAT TTA GAA CAG CTT TTG GGT GTC ATA GAA GTG AAC AAG TTT 962

286 K D L T Y L Y L E Q L L G V I E V N K F 305

963 GCT TTT GCA ACC CTA AAT CTA CGA GAG CTT TCA ATA GGA AAC ACT GTA AAG GGA TAC ATG 1022

306 A F A T L N L R E L S I G N T V K G Y M 325

1023 AAT GTG GTT GAT TTT TAT ACT TTT CTT CTT TAC AAT CCC TTT TTG AAA ATT CTA TCA CTG 1082

326 N V V D F Y T F L L Y N P F L K I L S L 345

1083 AAT AAC ATA AAT CTA TCT CTT GTA AAC ACA TCA AGG ATG CTT TCT AAG CTG ACT AAT CTA 1142

346 N N I N L S L V N T S R M L S K L T N L 365

1143 ACT TCC CTG TCT ATC CTA AAC TGT AAC ATA CAA AAC GTC CCC ACC TTA ACA AAT CCT CCA 1202

366 T S L S I L N C N I Q N V P T L T N P P 385

1203 TTG CTA TCG TCG ATT GAT ATG AGC TTT AAT TCA ATT CAG TCT CTG CAT GCT GAA GCC TTT 1262

386 L L S S I D M S F N S I Q S L H A E A F 405

1263 CAA TTC CTT GGC AAA TTA AAG TAT ATC TCT TTA AAG AGT AAT GCG CTT ACA TCA GTT ACA 1322

406 Q F L G K L K Y I S L K S N A L T S V T 425

1323 CTT GAA TCT TTA CCA AGG GTT CTC TGG GAT ACG AGT GAT GTT TTG ATT GAT CTC TCA TCA 1382

426 L E S L P R V L W D T S D V L I D L S S 445

1383 AAC CCC TTC GAT TGT GTC TGT AGT TTG GAG TGG TTC AAG TTT TGG TAT GAG AAC AAT ATG 1442

446 N P F D C V C S L E W F K F W Y E N N M 465

1443 AAA AGA ACA TCC GGC AAC GCC TCT CTG TAC ATA TGT AAC ACG CCC ACA CAA TGG CAG GAG 1502

466 K R T S G N A S L Y I C N T P T Q W Q E 485

1503 GTT CCC CTC GTC GAC TTT GAT ACC GCT GAC TGC CAT GAG CTG AAT CAA TAC GTG GTG ATG 1562

486 V P L V D F D T A D C H E L N Q Y V V M 505

1563 GCC CTT ATT ATA GCC GGT GAG TTA TGC CTT GTC GCA CTC TCC TTT ATC GTA GTC AGG ATA 1622

506 A L I I A G E L C L V A L S F I V V R I 525

1623 TAC CGA TGG GAT ATA AAG TAT TAC ATA CAT GCA TGC AAG TAC AAC AAA CTC ACT TCT CCG 1682

526 Y R W D I K Y Y I H A C K Y N K L T S P 545

1683 CCG GAA AAC TTG CGC GAC GAC TTC CTC TAC GAT GGC TTC GTC GCA TAC AAC ACA CGT GGC 1742

546 P E N L R D D F L Y D G F V A Y N T R G 565

1743 CGG AAA TGG ATC ATG GCG GAA CTG GTC GAA CAC GTA GAA AGG AAA CAT AAC TAC AAA CTC 1802

566 R K W I M A E L V E H V E R K H N Y K L 585

1803 TGT CTG CAC GAG AGA GAT ATC ATT CCA GGC GGG GTG TAT GTC GAA GAT GTT CTC GAA AGT 1862

586 C L H E R D I I P G G V Y V E D V L E S 605

1863 ATA GAT TTC AGT AGA AAG TTC ATA CTG GTT CTT TCG AAC AAC TTC ATG GAT GAC CAG TGG 1922

606 I D F S R K F I L V L S N N F M D D Q W 625

1923 GGC AGA TAC GAG ACA GCA ATT GCG AGT CAT ACT TTG GCG GAA GGG GGT GGT GGC AAA CTG 1982

626 G R Y E T A I A S H T L A E G G G G K L 645

1983 TTT CTC ATT TTA CTG GAA GAC ATC CGC TCT GAA TAC ATC ACC AGG TCC CTG AAG GTC TTG 2042

646 F L I L L E D I R S E Y I T R S L K V L 665

2043 CTG AAA TCT ATC AGA CAT GCT GAA TGG ACA AAA AAT AAA AAT GGA CAA AAA ATC TTC TGG 2102

666 L K S I R H A E W T K N K N G Q K I F W 685

2103 AAT AAC GTT GTA CAG AAT TTA GAA AAA TCC GAA AAA **TAA** TTGGGTTTTCTTTTTTCACAATTTAAA 2168

686 N N V V Q N L E K S E K * 697

2169 ATTTCTCTGTGGAATAACTAGTTTTGACATCCGCCACATTGTGACGCTAACCAATCGTGTTGTACGATAGAAGGAAATG 2246

2247 TTATGGTAAACA**AATAAA**CCATTAGGGCAAAAAAAAAAAAAAAAAA 2293

Myd88

1 TGACCCATGCCCCTGCCTTGGTTGGGGACGAGGCAGTGATGTAAGTGAGTAGGAACACCTTC 62

63 ATGACGATCCTGTCTTCCACTCACCGTATTTCTTCTATCCGAAACTAGGATTTTACGGGCGTGGGGACCTGGGGCGGGA 141

142 ATTGACAAGACCGACTCGTGGACGCAACTCACTGATATGTCCCATCGGATACACTGTATTATCGAACATAGGGAGCCAT 220

221 GGGCAGGTAATGAATTTCATAGTACCCTGTTTACTGATGGATTAAATCTGTGAATATCTGACTATACATCGCAATAAAA 299

300 **ATG** TCG ATC ACA TCG GAA CAG TTG ATT GTC GAG GGA AAA AAT GTC CCT CTT CAC GCC CTT 359

1 M S I T S E Q L I V E G K N V P L H A L 20

360 AAC ATG TCC GTC AGA TCA AAA CTA GGA ACT TAC CTA GAT CCG GAG GGT TTT GTA ACG GGA 419

21 N M S V R S K L G T Y L D P E G F V T G 40

420 GAC TAC TCC AAC GAT TAC CAA GGG CTG GCC GAG GTT ATC GGT TTC ACT TTC CAA GAC ATT 479

41 D Y S N D Y Q G L A E V I G F T F Q D I 60

480 ACA AAC TTC CAG AGA CAG AGT AAA CCG ACT CAG GAG ATG CTC TAT CAA TGG GGA ACG AGA 539

61 T N F Q R Q S K P T Q E M L Y Q W G T R 80

540 CCA GAA CTT TCA CCA ACG GTC GAC AAT CTC ATA AAA CAT TTG CAG CCG ATA GGG AGA TCA 599

81 P E L S P T V D N L I K H L Q P I G R S 100

600 GAT GAC GTT ATA ACA GAA TGC GCC CAT TTG ATA AAG AAA GAT GTG GAT CGA TAT AAG CAA 659

101 D D V I T E C A H L I K K D V D R Y K Q 120

660 TGC CAC AAA GAC ATT ACG GGC TCA AAA GAT ATG ATT CAG GAC CCT AGT GTT TCT CAA GGA 719

121 C H K D I T G S K D M I Q D P S V S Q G 140

720 CCA AAC CGA CCG TCC TGT GAC TAT GTT CCC GAG AGT GAC AAG TTA GGT CTA GTC ACC ATT 779

141	P N R P S C D Y V P E S D K L G L V T I	160
780	GAT GAC GTC AAA GAG AAA GGT GAC ACG CTC TAT TAC GAC GCC TTT GTG ATA TAC AAC CCT	839
161	D D V K E K G D T L Y Y D A F V I Y N P	180
840	GTG GGG AAG GAC TTG GAG TTC GTG AAG GAG CTC GCG GGG AAA ATG GAG GCC CCG CCT TAC	899
181	V G K D L E F V K E L A G K M E A P P Y	200
900	AAT CTA AAA TTC TGC ATC CCC TGG AGA GAC GAT CTG CCT GGA GGC TCT CGT TAT GAA GTC	959
201	N L K F C I P W R D D L P G G S R Y E V	220
960	TCT GCC CAC ATG ATT GCT ACA CGG TGT CGA AGA ACA CTG GTA ATC TTG TCT TCA GAT TTC	1019
221	S A H M I A T R C R R T L V I L S S D F	240
1020	TTA AAA AGT GCT GCG GCC GAT TTT CAG CTC AAA TTT GCC CAC TGT TTG TCA CCA GGT GCA	1079
241	L K S A A A D F Q L K F A H C L S P G A	260
1080	AGG AGT AAA AAA GTG GTC CCC GTA TTT TCC GCT CCG TGT AAA ATG CCG GGT ATT CTT CGA	1139
261	R S K K V V P V F S A P C K M P G I L R	280
1140	GCA GTG TCT TTC GTT GAT TTC ACG AAC CCC GGA TTA AGA GAC TGG AAC TGG CCC CGC CTT	1199
281	A V S F V D F T N P G L R D W N W P R L	300
1200	AAC GCT GTA TTA CGG TGC CCC CTA AAT CCC GAT CCC CGG GAT TAC ATG AGT GAG GCC GAA	1259
301	N A V L R C P L N P D P R D Y M S E A E	320
1260	CTT GAA GAA TTA AAA CTG AAC GCT GGG GTG ATT ACA AAG AGA ATG TGG TAC TCC ACT GGT	1319
321	L E E L K L N A G V I T K R M W Y S T G	340
1320	GTC CTA ACA ATG AAC TTC CCT GAA GAA TCC GAG GAC AAC ACT CCA TAC AAC GGC **TGA** GGG	1379
341	V L T M N F P E E S E D N T P Y N G *	358
1380	TGGCTAGTAGTTCTCGTGAAATGTATGGAGTAATGCAACACATTAATCCCATTGTAACGTTATTGAATACCGAGTTGAT	1458
1459	ATATCAACTTTGAATATTGCAACCTGATCAAACCTATTTTGTTAGGTTCTCCAAAAGACATATTATTTGTAACAATGTT	1537
1538	TCAGAAAGAAAACATTCGTA**ATTAAA**TTACGTCATCGTGAAAAAAAAAAAAAAAAAA	1594
