# Supplementary material for: Characteristic and Functional Analysis of Toll-like Receptors (TLRs) in the lophotrocozoan, Crassostrea gigas, Reveals Ancient Origin of TLR-Mediated Innate Immunity
Source: PLoS One. 2013 Oct 1;8(10):e76464. doi: 10.1371/journal.pone.0076464 (PMC3788107; doi:10.1371/journal.pone.0076464)
Supplement: Table S1 — Sequences of primers used in this study. “F” indicated forward primer, “R” indicated reverse primer. (DOCX) [file pone.0076464.s002.docx]

**Table 1.** Sequences of primers used in this study.

| **Primer** | **Sequence( 5’🡪 3’)** | **Comment** |
| --- | --- | --- |
| GR5P | CGACTGGAGCACGAGGACACTGA | 5’RACE first primer |
| GR5NP | GGACACTGACATGGACTGAAGGAGTA | 5’RACE nest primer |
| GR3P | GCTGTCAACGATACGCTACGTAACG | 3’RACE first primer |
| GR3NP | CGCTACGTAACGGCATGACAGTG | 3’RACE nest primer |
| TLR1-R1 | AAGGCATCTGGATCAACTTTGAACATCAC | 5’ RACE primer for TLR1 |
| TLR1-F1 | TAACAACTTGCATTCATTCAACGGTTTG | 3’ RACE primer for TLR1 |
| TLR1-R2 | TTTCACTTCCATCGCTGTTCGTC | 5’ RACE primer for TLR1 |
| TLR1-F2 | CCCGATCTTTGGAAATTGTCCCT | 3’ RACE primer for TLR1 |
| TLR2-R1 | ACAAACTTCGTCCAGTCGCATCG | 5’ RACE primer for TLR2 |
| TLR2-F1 | CTCTTTGGTCGAAGTCCGATGCG | 3’ RACE primer for TLR2 |
| TLR2-R2 | TATCCTCATCGGCATAAACTCCC | 5’ RACE primer for TLR2 |
| TLR2-F2 | TAATGGTCTGCTCGGTGTTCTCC | 3’ RACE primer for TLR2 |
| TLR3-R1 | TTCTTTCCGAACCCATGCACAAT | 5’ RACE primer for TLR3 |
| TLR3-F1 | TGGTATCCTAGTGCTTGTCGTCTGCGTAA | 3’ RACE primer for TLR3 |
| TLR3-R2 | CTGAACCATCGGAGGTCGCAAGT | 5’ RACE primer for TLR3 |
| TLR3-F2 | CATCAATGTTGGTGGTGTTCCGTAT | 3’ RACE primer for TLR3 |
| TLR4-R1 | GGCGGAGAAGTGAGTTTGTTGTACTTGCA | 5’ RACE primer for TLR4 |
| TLR4-F1 | ATTGCGAGTCATACTTTGGCGGAAGG | 3’ RACE primer for TLR4 |
| TLR4-R2 | GCATAACTCACCGGCTATAATAAGG | 5’ RACE primer for TLR4 |
| TLR4-F2 | CACCAGGTCCCTGAAGGTCTTGC | 3’ RACE primer for TLR4 |
| Myd88-F1 | TGCAACACATTAATCCCATTGTAACG | 3’ RACE primer for MyD88 |
| Myd88-F2 | TGCAACCTGATCAAACCTATTTTGTT | 3’ RACE primer for MyD88 |
| Myd88-R1 | CCTTGCACCTGGTGACAAACAGTGGG | 5’ RACE primer for MyD88 |
| Myd88-R2 | TTTGAGCTGAAAATCGGCCGCAGCAC | 5’ RACE primer for MyD88 |
| TLR1-F | GTGACGAACAGCGATGGAAG | Real-time PCR for TLR1 |
| TLR31-R | TGGGCAATGAAGGAAAGTAA | Real-time PCR for TLR1 |
| TLR2-F | TTGGGAAGAAATAAGCTACAAG | Real-time PCR for TLR2 |
| TLR2-R | AACTTCGTCCAGTCGCATCG | Real-time PCR for TLR2 |
| TLR3-F | AGTGCTTGTCGTCTGCGTAA | Real-time PCR for TLR3 |
| TLR3-R | ATCGTCCTCATCTGCATTGGAT | Real-time PCR for TLR3 |
| TLR4-F | CGGTGAGTTATGCCTTGTCG | Real-time PCR for TLR4 |
| TLR4-R | CGAAGCCATCGTAGAGGAAGT | Real-time PCR for TLR4 |
| GAPDH-QF | TCACTGCTACCCAGAAGACCG | Real-time PCR for GAPDH |
| GAPDH-QR | CTCAGGAATGACCTTGCCCAC | Real-time PCR for GAPDH |
| EF1α-QF | GACAAACTGAAGGCAGAGCG | Real-time PCR for EF1α |
| EF1α-QR | CAGCCTGTGAAGTTCCTGTAAT | Real-time PCR for EF1α |
| Rpl13-QF | TCCTCCCTGCATCAGTAATT | Real-time PCR for rpl13 |
| Rpl13-QR | CCAAGAAACAGAGCAAACCC | Real-time PCR for rpl13 |
| Myd88-qF | TGGGGAACGAGACCAGAAC | Real-time PCR for MyD88 |
| Myd88-qR | TCTTTGTGGCATTGCTTAT | Real-time PCR for MyD88 |
| TLR1-f3 | GTGCAAGCTTATGGAAGTGAAAATAACAGTT | pcdna4.0 Vector for TLR1 |
| TLR1-R3 | GTGTGATATC T AATAGCAATTGGTGGATTACC | pcdna4.0 Vector for TLR1 |
| TLR2-f3 | GTGCGGATCCATGACCAGAGCACAGATTTT | pcdna4.0 Vector for TLR2 |
| TLR2-R3 | GTGTCTCGAGTTGGAGAACACCGAGCAGAC | pcdna4.0 Vector for TLR2 |
| TLR3-f3 | GTGCGGATCCATGAAGATGCATACTACTCCA | pcdna4.0 Vector for TLR3 |
| TLR3-R3 | GGTCCTCGAGTACAACGACATGTTTAGTTATC | pcdna4.0 Vector for TLR3 |
| TLR4-f3 | GTGCGGATCCATGAAGTATTTACGGACGTTG | pcdna4.0 Vector for TLR4 |
| TLR4-R3 | GGTCCTCGAGTTTTTCGGATTTTTCTAAATTC | pcdna4.0 Vector for TLR4 |
| TLR1-tirF | CTCTGGATCCAAGAAGCGATGGTGGATTCT | pCMV-n-ha Vector for TLR1-tir |
| TLR1-tirR | ACACGAATTCTTATACAACGACATGTCTAG | pCMV-n-ha Vector for TLR1-tir |
| Myd88-F3 | GTGCGGATCC AAAATGTCGATCACATCGGAAC | pcdna4.0 Vector for MyD88 |
| Myd88-R3 | GTGTCTCGAG GCCGTTGTATGGAGTGTTGT | pcdna4.0 Vector for MyD88 |
| Myd88-F5 | GTGTGGATCCGATGTGGATCGGTATAAGCA | pCMV-N-flag Vector for MyD88-tir |
| Myd88-R5 | CTCTCTCGAGTCAGGTGACTAGACCTAACTTGTC | pCMV-N-flag Vector for MyD88-tir |
| TLR1-F4 | GTGCAAGCTTATGGAAGTGAAAATAACAGTT | pEGFP Vector for TLR1 |
| TLR1-R4 | GTGTGTCTACTGAATAGCAATTGGTGGATTACC | pEGFP Vector for TLR1 |
| TLR2-F4 | GTGCCTCGAGATGACCAGAGCACAGATTTT | pEGFP Vector for TLR2 |
| TLR2-R4 | GTGTGGATCCCGTTGGAGAACACCGAGCAGAC | pEGFP Vector for TLR2 |
| TLR3-F4 | GTGCAAGCTTATGAAGATGCATACTACTCCAGG | pEGFP Vector for TLR3 |
| TLR3-R4 | TCGGATCCCGTACAACGACATGTTTAGTTATC | pEGFP Vector for TLR3 |
| TLR4-F4 | GTGCAAGCTTATGAAGTATTTACGGACGTTG | pEGFP Vector for TLR4 |
| TLR3-R4 | GGTCGGATCCCGTTTTTCGGATTTTTCTAAATTC | pEGFP Vector for TLR4 |
