# Supplementary material for: Characteristic and Functional Analysis of Toll-like Receptors (TLRs) in the lophotrocozoan, Crassostrea gigas, Reveals Ancient Origin of TLR-Mediated Innate Immunity
Source: PLoS One. 2013 Oct 1;8(10):e76464. doi: 10.1371/journal.pone.0076464 (PMC3788107; doi:10.1371/journal.pone.0076464)
Supplement: Table S2 — Sequences used for MDS analysis. (DOCX) [file pone.0076464.s003.docx]

**Table S2.** Sequences used for MDS analysis.

| **Classification** | **TLR proteins and their accession numbers** |
| --- | --- |
| **Vertebrate TLRs** | Danio rerio TLR1, NP_001124065  Danio rerio TLR13, XP_002664893  Danio rerio TLR2, NP_001082819  Danio rerio TLR2, NP_997977  Danio rerio TLR21, NP_001186264  Danio rerio TLR22, NP_001122147  Danio rerio TLR3, NP_001013287  Danio rerio TLR4, NP_997978  Danio rerio TLR7, XP_003199309  Danio rerioTLR9, NP_001124066  Gallus gallus TLR1, BAD67422  Gallus gallus TLR2, BAB16843  Gallus gallus TLR21, NP_001025729  Gallus gallus TLR3, ADZ48550  Gallus gallus TLR4, NP_001025864  Gallus gallus TLR5, ACR26254  Gallus gallus TLR7, NP_001011688  Gallus gallus, TLR15, NP_001032924  Homo sapiens TLR 7, NP_057646  Homo sapiens TLR1, NP_003254  Homo sapiens TLR10, NP_112218  Homo sapiens TLR2, NP_003255  Homo sapiens TLR3, ABC86908  Homo sapiens TLR4, NP_612564  Homo sapiens TLR5, NP_003259  Homo sapiens TLR6, NP_006059  Homo sapiens TLR8, NP_619542  Homo sapiens TLR9, EAW65192  Takifugu rubripes TLR 7, XP_003961920  Takifugu rubripes TLR1, AAW69368  Takifugu rubripes TLR2, AAW69369  Takifugu rubripes TLR2, AAW69370  Takifugu rubripes TLR21, BAC66138  Takifugu rubripes TLR22, AAW69372  Takifugu rubripes TLR22, AAW70378  Takifugu rubripes TLR3, AAW69373  Takifugu rubripes TLR5, AAW69374  Takifugu rubripes TLR8, XP_003961919  Takifugu rubripes TLR9, XP_003963351 |
| **Invertebrate TLRs** | Aedes aegypti toll, XP_001658507  Apis mellifera toll, XP_396158  Azumapecten farreri Toll receptor, ABC73693  Branchiostoma belcheri TLR13, ABD58972  Caenorhabditis elegans toll, AAK37544  Crassostrea gigas TLR1, KC700617  Crassostrea gigas TLR2, KC700618  Crassostrea gigas TLR3, KC700619  Crassostrea gigas TLR4, KC700620  Drosophila melanogaster 18 wheeler, NP_476814  Drosophila melanogaster MstProx, NP_649719  Drosophila melanogaster TLR6, NP_524081  Drosophila melanogaster TLR7, NP_523797  Drosophila melanogaster TLR8, NP_524757  Drosophila melanogaster Toll-4, NP_523519  Drosophila melanogaster Toll-5, AAF86227  Drosophila melanogaster Toll-9, NP_649214  Euprymna scolopes TLR, AAY27971  Mytilus galloprovincialis TLRj, AGG10807  Strongylocentrotus purpuratus TLR1, AAK21261  Strongylocentrotus purpuratus TLR2, NP_999671  Strongylocentrotus purpuratus TLR3, NP_999670  Tachypleus tridentatus toll, BAD12073  Tribolium castaneum toll-like, XP_967796 |
